# Microbiome-based enrichment pattern mining has enabled a deeper understanding of the biome–species–function relationship

**DOI:** 10.1038/s42003-023-04753-x

**Published:** 2023-04-10

**Authors:** Pengshuo Yang, Xue Zhu, Kang Ning

**Affiliations:** 1 Key Laboratory of Molecular Biophysics of the Ministry of Education, Hubei Key Laboratory of Bioinformatics and Molecular-imaging, Center of AI Biology, Department of Bioinformatics and Systems Biology, College of Life Science and Technology, Huazhong University of Science and Technology, Wuhan, 430074 China; 2 Institute of Medical Genomics, Biomedical Sciences College, Shandong First Medical University, Shandong, 250117 China

**Keywords:** Microbial ecology, Microbial ecology, Evolutionary theory

## Abstract

Microbes live in diverse habitats (i.e. biomes), yet their species and genes were biome-specific, forming enrichment patterns. These enrichment patterns have mirrored the biome–species–function relationship, which is shaped by ecological and evolutionary principles. However, a grand picture of these enrichment patterns, as well as the roles of external and internal factors in driving these enrichment patterns, remain largely unexamined. In this work, we have examined the enrichment patterns based on 1705 microbiome samples from four representative biomes (Engineered, Gut, Freshwater, and Soil). Moreover, an “enrichment sphere” model was constructed to elucidate the regulatory principles behind these patterns. The driving factors for this model were revealed based on two case studies: (1) The copper-resistance genes were enriched in Soil biomes, owing to the copper contamination and horizontal gene transfer. (2) The flagellum-related genes were enriched in the Freshwater biome, due to high fluidity and vertical gene accumulation. Furthermore, this enrichment sphere model has valuable applications, such as in biome identification for metagenome samples, and in guiding 3D structure modeling of proteins. In summary, the enrichment sphere model aims towards creating a bluebook of the biome–species–function relationships and be applied in many fields.

## Abstract (web summary)

Enrichment sphere modeling indicates that both microbial species and functional gene enrichment is evident within different biomes

## Introduction

Microbes, being pervasive and important organisms in nature, microbes often dwell as a microbial community in a variety of habitats (i.e. biomes). Much effort has been made to investigate the “biome–species” relationship, which revealed an uneven species distribution across biomes^1,2^ in response to the biome-specific environmental stresses^3–5^. For example, Mallott et al. reported a host-specific distribution for species in the gut microbiome^6^, while Thompson et al. discovered that in the global soil microbiome, the mid-latitude area was identified with the greatest diversity of species^2^. With a deeper insight into the microbial community, it would be reasonable to ask: what factors are important for defining the distributions of species and genes within and across various biomes? Previous research has revealed that environmental factors and gene communication events, especially Horizontal Gene Transfer (HGT), are the most important factors influencing the microbial community^7,8^. To systematically illustrate the relationship between environmental factors and microbial communities, a gene ontology-like hierarchical annotation, named biome, was presented. The biome’s top-level structure is classified into Host-associated, Environmental, and Engineered, and the MGnify database has thus far retrieved 491 biomes^9^.

There are diverse microbial compositions in various biomes, and to respond to environmental pressure, the functional distribution of microbes will shift to help their host adapt to this pressure^10,11^. The functional genes that drive the biome–species relationship, also showed a species-specific distribution, and properly leveraging the “species–function” relationship could lay the groundwork for determining the host’s functional responsibilities in a microbial community:^12–14^ Many research has revealed that gene functions in microbial communities are critical for their hosts’ responses to environmental pressure (e.g., the different contents of salinity, temperature, oxygen and total nitrigon), collectively referred to as external factors^15,16^. For example, *Bacteroides*, which colonizes a wide range of environments, is responsible for transcribing different functional genes to degrade biome-specific organic compounds^17,18^.

In addition, gene communication across the microbial community is essential for the dispersal of species and genes across diverse biomes^19,20^. As a response to a changed environment, members would transfer their genes through different approaches, including horizontal gene transfer^21^, vertical gene accumulation^22^, resource competition^23^, and nutrient cross-feeding^24^, which were defined as the internal factors in microbial communities. These internal factors play major roles in the rapid sharing of functional genes^21,25^ and can result in alterations to the host genome^20,26^, which provide a selective advantage to microbes in their living biome^21^. With the advent of metagenomic sequencing, we can detect all the nucleic acid sequences of the microbial community, enabling the detection of gene communications. Based on the features of evolutionary location, base composition, selection pressure mutation, etc^19,21^, numerous bioinformatic tools, such as MetaCHIP^27^, and MGEfinder^28^ have been developed. Despite the extraordinary significance that these methods have had in recent extensive attempts to research the biome–species and species–function relationships, internal factors in microbial communities remain largely unexplored.

Recent studies have emphasized the combination of biome–species and downstream species–function relationships toward the establishment of the “biome–species–function” relationship, as well as to deduce complex patterns and prospective applications in microbiome^29^. For example, Hou et al. collected the microbiome from a deep-sea hydrothermal vent that has an abnormally high sulfide concentration^30^, and they have identified two novel genera *Campylobacteria* and *Aquificae*, whose dominance in the community is driven by two dissimilatory sulfate reduction genes: *aprA/B* and *dsrA/B*. Another illustration is about leveraging biome–species–function relationship to assist in modeling the protein 3D structures^31^. Wang et al. utilized 97 million proteins from the global ocean microbiome to supplement the homologous sequences for proteins with unknown structures and successfully predicted the structures for 12 protein families, which were prevalent and exert important functions in marine microbiome^31^. These findings demonstrated the critical need for a full understanding of the biome–species–function relationship.

Herein, we deciphered the biome–species–function relationship on a systemic level, exploring the external and internal factors that contribute to this relationship. By integrating the enrichment analysis with the ecology and evolution analysis of microbial community, this relationship was depicted as an “enrichment sphere” model, with its biological applications elucidated. We collected 1705 metagenome samples from four representative biomes (second layer in MGnify database: Engineered, Gut, Freshwater, and Soil)^32^, to discover the gene and species enrichment patterns across biomes. We found that the species and gene distributions were biome-specific. By concentrating on decoding the biome–species–function relationship using the enrichment analysis, the results mirrored an “enrichment sphere” model: different biomes have enriched different sets of species and functional genes (“enrichment” phenomenon), whereas genes with similar functions and their hosts would be enriched within the ontologically adjacent biomes, forming a “sphere”. Moreover, combining the analysis of the external and internal factors driving these enrichment patterns, our research provides a deeper understanding of the evolution and ecology law of the microbial community through two case studies: (1) The copper-resistance genes and their hosts were enriched in Soil biomes, which were driven by copper contamination and horizontal gene transfer. (2) The flagellum-related genes and their hosts were enriched in Freshwater biomes, due to high fluidity and vertical gene accumulation. These two case studies demonstrated prevalent strategies for gene dissemination through vertical accumulation and horizontal transformation to influence the biome–species–function relationship. Furthermore, by utilizing genes and species which have shown enrichment patterns, we could accurately identify the microbial community’s habitat biomes. Finally, we explored the biological application of the enrichment sphere model for homologous gene mining toward *de novo* protein 3D structure modeling. In summary, we decoded the biome–species–function relationship that was driven by both external and internal factors, which has been mirrored by the enrichment sphere model. Our work also emphasized the potential of this model for mechanism discovery and concrete applications.

## Results

### Profiling of microbiome samples to characterize the biome–species relationship

**Fig. 1:**
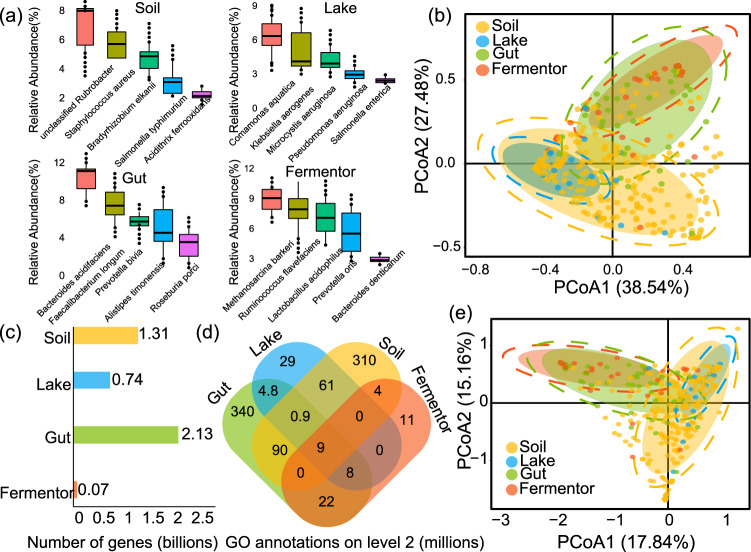
Taxonomical composition and functional profiles of microbiome samples from four biomes (Engineered, Gut, Freshwater, and Soil). **a** The top five species in each of the four biomes are sorted by average relative abundance. **b** PCoA result for samples from the four biomes based on taxonomical compositions at the species level. **c** Numbers of functional genes (billions) in the four biomes. **d** The common and unique functional distributions for the four biomes. The number labeled in the figure means the number (in millions) of specific or sheared genes annotated by the GO database on gene ontology (level 2). **e** PCoA results based on the functional profile of samples from the four biomes. In **b** and **e**, a point means a metagenome sample, and samples from the same biome are labeled with the same color. Circles indicate the confidence intervals for samples from the same biome.

Based on the high-quality of raw reads and assembled contigs (Supplementary Fig. S1), taxonomical analysis of 1705 samples from the four representative biomes (Engineered: 141, Gut:1,318, Freshwater: 66, and Soil:180) revealed that four biomes were identified with divergent species distributions (Fig. 1a, Supplementary Fig. S2) and species diversity among different samples (Supplementary Fig. S3). Additionally, the principal coordinate analysis (PCoA) based on taxonomical compositions at the species level indicated a biome-specific pattern (Fig. 1b): samples collected from the same biome were clustered into the same group (reflected by a concentrated confidence circle), while samples collected from different biomes were clustered into different groups (represented by the condensed confidence circle for different biomes). These results implied an intuitive view of the biome-specific species distributions across biomes.

Similar to the taxonomical composition, the functional profile exhibited a biome-specific pattern. First, different biomes with variable numbers of functional genes were identified (Fig. 1c): Soil: 1.31 billion, Freshwater: 0.74 billion, Gut: 2.13 billion, and Engineered: 0.07 billion (Supplementary Fig. S4). Second, the Gene Ontology (GO) database was utilized to further integrate the functional genes, and the four biomes were also identified with different counts of GO annotations (Soil: 520 million, Freshwater: 120 million, Gut: 1,530 million, and Engineered: 67 million) (Fig. 1d and Supplementary Fig. S5). Finally, the PCoA results based on GO annotation indicated a different functional profile across the four biomes (Fig. 1e). Using the functional annotations provided by HUMAnN 2 (Supplementary Fig. S6), the biome-specific distribution was also identified. In conclusion, profiling of microbiome samples characterized the biome–species–function relationship: both taxonomical compositions and functional profiles illustrated biome-specific patterns, which were unique across biomes^33^. This finding establishes biogeographical distribution patterns for biome–species–function relationships across biomes.

### The enrichment sphere model

**Fig. 2:**
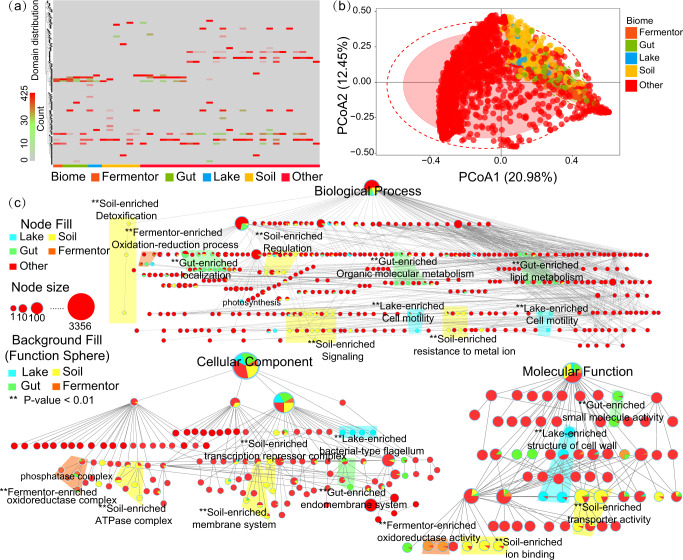
Functional gene enrichment in biomes as determined by GO annotation term analysis. In **a** and **b**, the label of samples was assigned based on the result of the enrichment analysis. The term “other” refers to protein domains that are not significantly enriched (*P*-value > 0.5) in any of the four biomes. **a** Protein domain enrichment in four biomes. The heatmap illustrates the distribution of protein domains according to their enriched biomes. Each row means a protein domain and each column means a metagenome sample, grouped by its biome. **b** PCoA results for samples from the four biomes based on the protein domain distribution. Samples from the same biome are labeled with the same color. Circles indicate the confidence intervals for samples from the same biome. **c** The enrichment of functions in the four biomes based on a cluster of GO annotations. The proportions of the four biomes in a GO annotation are labeled on corresponding GO annotations in pie chart form. A cluster of GO annotations enriched in a specific biome is annotated by a colored polygon in the background fill to represent this enrichment pattern, one color for each biome. The enriched sphere was labeled with “**” for *P*-value < 0.01. And representative clusters are also annotated by text, for example, “Soil-enriched ion binding”. The entire procedure for the building of the enrichment sphere model is described in “Materials and methods”.

Due to the uneven distribution of species and genes across biomes, it is reasonable to assume that species and genes in the microbial community would exhibit different enrichment patterns to adapt to their habitat biomes. Our enrichment analyses revealed that many species and genes had significantly different abundances in one biome compared to others, rather than being uniformly distributed throughout four biomes. And such uneven distributions of species and genes are prevalent throughout biomes. First, a landscape of functional gene distributions for the four biomes was created by annotating the protein domains, resulting in 6,415 divergent protein domains for the four biomes (Soil: 2,914; Freshwater: 987; Gut: 2,011; Engineered: 503). Further enrichment analysis demonstrated that domains with similar functions were enriched in specific biomes to help their host adapt to their biome (Fig. 2a). For example, the Freshwater biome enriched with the protein domains PSII_BNR (301 counts, *P*-value = 6.25e–8) and PRK13684 (251 counts, *P*-value = 4.25e–6). Both are photosynthesis-related protein domains, which were important for the Freshwater microbiome^34,35^. The PCoA analysis also indicated a biome-specific enrichment pattern for protein domains (Fig. 2b). Taken as a whole, this biome-specific gene distribution necessitated an enrichment analysis to uncover significant differences across the four biomes.

A deep understanding of the biome–species–function relationship has spawned an “enrichment sphere” model. Firstly, we determined the functional genes or species with significantly higher abundance in one biome than in others^36^. GO was annotated with a hierarchical structure for recording the relationship among multiple annotations, which is more favorable to mechanism research and pattern mining than other functional annotations. Thus, we mapped functional genes to the GO database and retrieved 845 GO annotations (biological process: 421; cellular component: 284, and molecular function: 140). We found enrichment patterns for species and genes were prevalent. Taking the species *Bacteroides acidifaciens* as an example, this species was enriched in the Gut biome (*P*-value = 3.25e–15) to help their host in degrading dietary fibers in the gut^37^. Another example GO:0055114 (biological process, oxidation-reduction process) was significantly enriched in Engineered (*P*-value = 6.24e–12), potentially due to its distinctive and primary function in the Engineered^38^. Secondly, we combined the enriched species and enriched GO annotations by testing whether the genes that were associated with enriched host species within a biome share similar functions. Different sets of GO enrichments and their gene hosts were identified in each of the four biomes: 32 GO annotations and 66 gene hosts for the Soil biome, 11 GO annotations and 28 gene hosts for the Freshwater biome, 9 GO annotations and 22 gene hosts for Gut biome, 10 GO annotations and 12 gene hosts for the Engineered biome. Thirdly, we found that enriched GO annotations tend to be adjacent to the GO ontology (Supplementary Fig. S7). We performed enrichment analysis on GO annotations considering their neighboring nodes (ontologically adjacent or their parent nodes in the GO annotations, which means they are identified with similar function^39^), which revealed that similar functions of genes were enriched in similar biomes, forming a function sphere, and we also discovered that this is a prevalent phenomenon. Finally, the “enrichment sphere” model (Fig. 2c and Table 1) was constructed to combine these function spheres containing enriched GO annotations and their hosts.

**Table. 1:** Representative enrichment patterns in the enrichment sphere model for biome-species-function relationship.

GO classification	Function sphere	Enriched GO annotation^a^	Enriched host species^b^
***Soil***			
Biological process	Detoxification	GO:1990748, GO:0071722, GO:0140725	*Glycera nicobarica*, *Planctomycetia bacterium*, *Pedosphaera parvula*
Biological process	Regulation of biological process	GO:0044145, GO:0048519, GO:0048518	*Paenibacillus sp.27-9*, *Verrucomicrobia bacterium SCGC AG-212-E04*, *Bacterium Ellin5102*
Biological process	Signaling	GO:0007267, GO:0035426, GO:0021807	*Staphylococcus aureus*, *Salmonella typhimurium*, *Acidithrix ferrooxidans*
Biological process	Resistance to metal ion	GO:0071248, GO:0010044, GO:0046686	*Staphylococcus aureus ET3* − *1*, *Staphylococcus aureus USA300*, *Acidithrix ferrooxidans*
Celluar Component	ATPase complex	GO:0062091, GO:1904564, GO:0070603	*Enterococcus hirae*, *Yersinia pestis*, *Quercus lobata*
Celluar Component	Membrane system	GO:0005642, GO:0036362, GO:0048475	*Bradyrhizobium elkanii, bacterium Ellin5102*, *Bacteroidetes_bacterium_N2*
Celluar Component	Transcription repressor complex	GO:1990512, GO:0036411, GO:0090570	*Salmonella typhimurium*, *Sphingobacteriales bacterium UTBCD1*, *gamma proteobacterium W1.09-152*
Molecular Function	Ion binding	GO:0043167, GO:0043168, GO:0043169	*Staphylococcus sp. SAU*, *Actinobacterium YJF1-30*, *Segetibacter koreensis*
Molecular Function	Transporter activity	GO:0005319, GO:0032410, GO:0032411	*Bacterium Ellin5102*, *Bacteroidetes bacterium N2*, *Segetibacter aerophilus*
***Freshwater***			
Biological process	Cell motility	GO:0070358, GO:0071976, GO:0016477	*Escherichia coli*, *Kosakonia radicincitans*, *Edwardsiella tarda*
Biological process	Photosynthesis	GO:1905156, GO:0019685, GO:0019684	*Comamonas aquatica*, *Oleispira antarctica*, *Alteromonas macleodii*
Celluar Component	Bacterial-type flagellum	GO:0009425, GO:0009420, GO:0009424	*Rhodobacter flagellatus*, *Pelagibacteraceae bacterium ETNP-OMZ-SAG-A7*, *Prevotella copri*
Molecular Function	Structural of cell wall	GO:0005198, GO:0005199, GO:1990915	*Phaeocystis antarctica*, *Klebsiella aerogenes*, *Pseudomonas aeruginosa*
***Gut***			
Biological process	Localization	GO:0051641, GO:0036214, GO:0051234	*bacterium NLAE-zl-H174*, *Akkermansia muciniphila*, *Bacteroides acidifaciens*
Biological process	Lipid metabolism	GO:0044255, GO:1900555, GO:1901568	*Faecalibacterium longum*, *Prevotella bivia, Roseburia porci*
Biological process	Organic molecular metabolism	GO:1901440, GO:1902061, GO:0042197	*bacterium NLAE-zl-H174*, *Akkermansia muciniphila*, *Prevotella bivia*
Celluar Component	Endomembrane system	GO:0005905, GO:0005783, GO:0005768	*Clostridium bolteae*, *Roseburia faecis*, *Bacteroides vulgatus*
Molecular Function	Small molecule activity	GO:0097063, GO:0061891, GO:0070027	*bacterium NLAE-zl-H174*, *Akkermansia muciniphila*, *Bacteroides sp*.
***Engineered***			
Biological process	Oxidation-reduction process	GO:0006725, GO:0046483, GO:1901360	*Pseudomonas moraviensis*, *Methanosarcina barkeri*, *Flavobacteriaceae bacterium_UJ101*
Celluar Component	Phosphatase complex	GO:1904097, GO:0106095, GO:1904144	*Bifidobacterium longum*, *Bacteroides fragilis*, *Prevotella oryzae*
Molecular Function	Oxidoreductase activity	GO:0018699, GO:0050697, GO:0018702	*Pseudomonas moraviensis*, *Ruminococcus flavefaciens*, *Flavobacteriaceae bacterium UJ101*

In each of the four biomes, the identified enrichment sphere was listed. The representative GO annotations and their representative enriched host in the corresponding function sphere were also listed. A detailed list of the enriched GO annotation and enriched host species was provided in Supplementary Data 1. Only GO terms with significant enrichment (*P*-value < 0.01), and species with significant enrichment (*P*-value < 0.01), were listed.

^a^Top three enriched GO annotation in function sphere, ranked by the number of GO annotations.

^b^The top three enriched species for the host of enriched GO annotations, listed according to their relative abundance within the biome.

Additionally, the enrichment sphere model is highly interpretable. For example, based on the enrichment sphere model (Fig. 2c and Table 1), the Soil biome enriched genes and their hosts related to resisting metal ions (biological process) and transporter activity (molecular function) to adapt to heavy metal contaminations^40,41^. Another example is that the functional genes and their hosts associated with cell motility (biological process; Fig. 2c and Table 1), were enriched in the Freshwater biome, although such an enrichment pattern would help their host to adapt to the fluid environment^42,43^.

Collectively, we proposed an enrichment sphere model that reflects the biome–species–function relationship: genes with similar functions, as well as their hosts, were enriched in specific biomes, forming a sphere of enrichment (enriched GO annotations for genes and their hosts are listed in Supplementary Data 1), and these functional genes and their hosts also dominated in their corresponding biomes (Supplementary Figs. S8–S11). This enrichment sphere model has charted a clear picture of the biogeography of species and functional genes in the presence of various influence factors.

### The enrichment sphere model reveals the species enriched with copper-resistance genes to resist copper contamination in the Soil biome

**Fig. 3:**
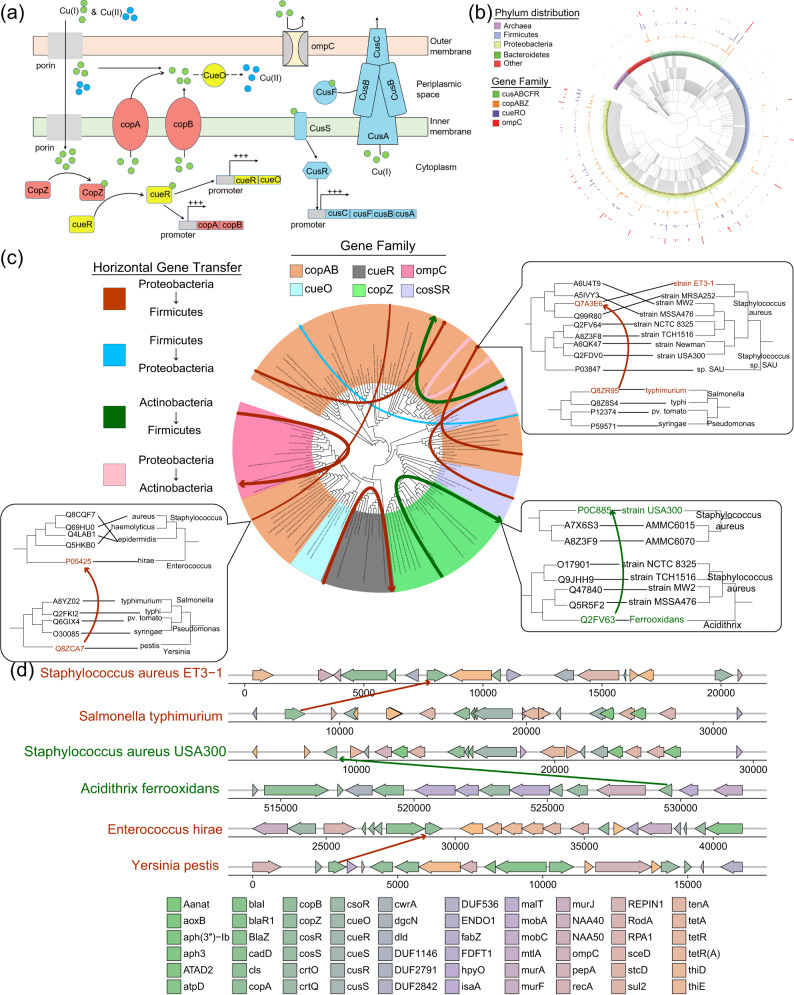
The species enriched with copper-resistance genes in Soil biomes. **a** The copper resistance mechanism in bacteria. Numerous genes cooperate to degrade or extrude the copper to protect their host. The direction of the arrow denotes the flow direction of copper ions. **b** A phylogenetic of species detected with copper resistance genes, including archaea and bacteria. The four outrings represent the gene counts for matching genes in distinct biomes. The lengths of outer rings represent the number of given gene families in this species. **c** Horizontal gene transfer events across different phyla using copper resistance genes as mobile elements. Different gene transfer events are labeled with different colors. The top three events ordered by *P*-value were presented in detail. For the phylogenetic tree, the tree on the left was constructed based on the copper proteins labeled with UniProtKB id. The tree on the right was constructed by the phylogenetic relationship of its hosts. **d** Top three gene transfer events based on the copper resistance genes. Each arrow corresponds to a gene, and the color of the arrows corresponds to the gene’s name. The genome was selected based on the result in (**c**) with the same color. The arrow across different species means the horizontal gene transfer events. For example, the brown arrow means the gene “*copA*” was transferred from the species *Yersinia pestis* to *Enterococcus hirae*.

The enrichment sphere model also allowed us to comprehend the internal factors, especially for horizontal gene transfer (HGT) events, which is the one of most important events in influencing microbial communities^20,26^. Combined the HGT detection result (MGEfinder, in sensitive mode^28^) with a literature search for genes within enriched GO annotation, this model provided a deep understanding of how copper resistance evolved into a fully developed life history strategy for species in the Soil biome (Fig. 3). Copper, as a major soil environmental stress, may harm soil microbiomes^44,45^. As a complex function, a full set of genes is involved to degrade or extrude copper in the Soil biome (Fig. 3a). In our metagenome dataset, 11 copper resistance genes were identified (Fig. 3b), mainly in four phyla: Proteobacteria (458 counts), Thaumarchaeota (228 counts), Firmicutes (189 counts), and Bacteroidetes (164 counts), which are all dominant phyla in Soil biomes (relative abundance: 13.5%, 4.28%, 6.96%, and 10.25%, respectively). Based on the enrichment sphere model (Fig. 2c and Table 1), these genes and their hosts were enriched in the Soil biome (Supplementary Data 1, Supplementary Fig. S12). Interestingly, six HGT events involving copper-resistant genes as mobile elements were found (Fig. 3c). The species involved in these HGT events were enriched in the Soil biome, and a full set of the copper-resistance gene could be detected in their host genome (Fig. 3c), which was in line with previous studies^25,46^. These results could help to explain why these genes and their hosts were enriched in the Soil biome: The frequent HGT events involving copper-resistance genes for these species attest to their predominance under environmental stresses from copper contamination. Under the effect of Copper genes, species that recognized HGT events would cluster with one another rather than with their phylogenetic neighbors (Supplementary Fig. S13).

### The enrichment sphere model reveals the species enriched with flagellum-related genes to gain a survival advantage in Freshwater biomes

**Fig. 4:**
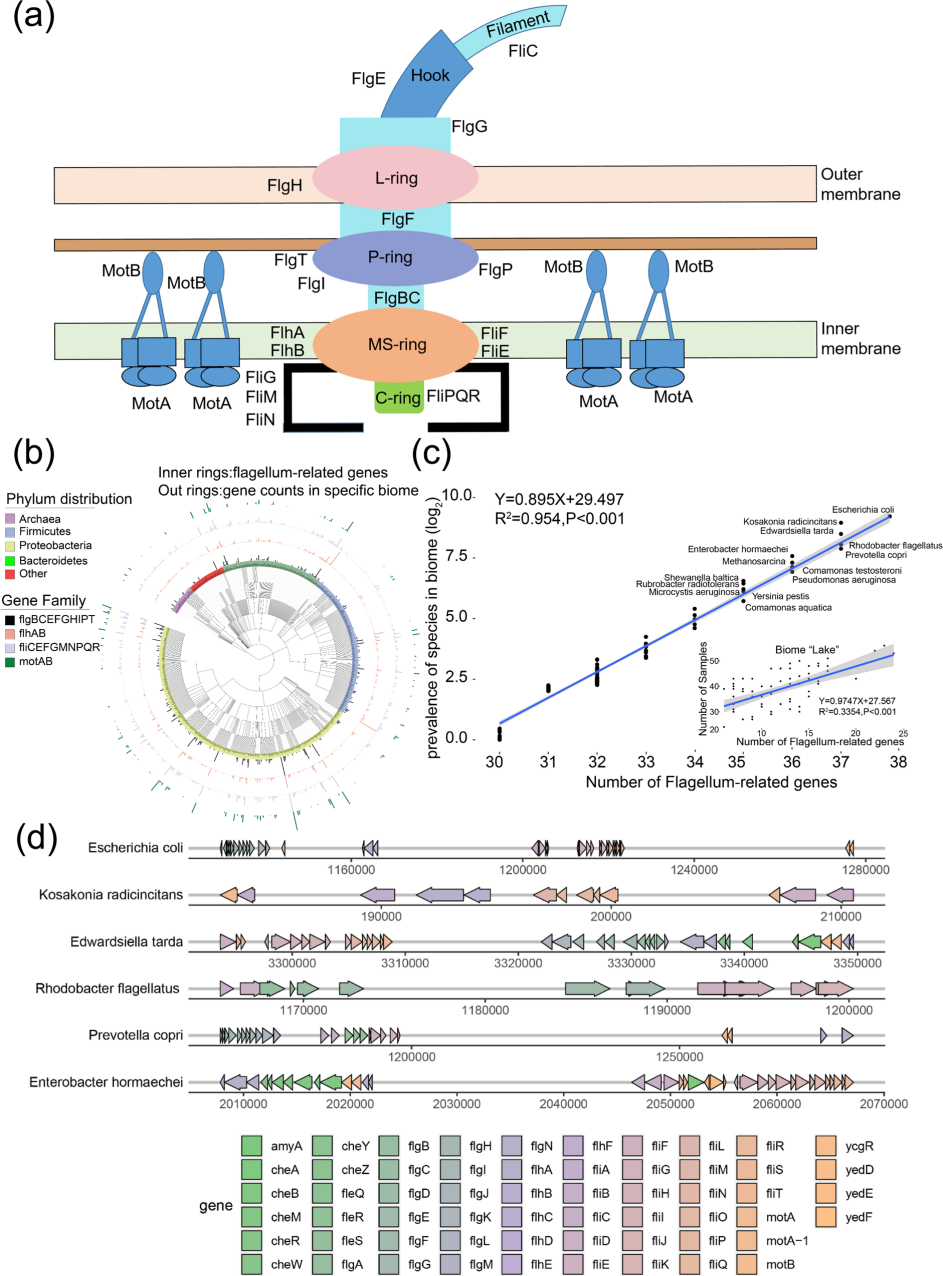
The species enriched with flagellum-related genes in Freshwater biomes. **a** The gene is involved in the development of bacteria’s flagellum. **b** A phylogenetic of species detected with flagellum-related genes, including archaea and bacteria. The four outrings represent the gene counts for matching genes in distinct biomes. The lengths of outer rings represent the number of given genes in this species. **c** Correlation between the number of flagellum-related genes and the prevalence of the host species in Freshwater biome. Each node represents a species, and the *X*-axis represents the number of flagellum-related genes in that species. The *Y*-axis means the counts of species in the Freshwater biome. The sub-figure in the bottom right corner of (**c**) depicts the correlation between the proportion of samples containing a certain species (*Y*-axis) and the number of flagellum-related genes found in the Freshwater biome. **d** The top five species ranked by the number of detected flagellum-related genes. The genome was selected based on the result in (**c**). Each arrow corresponds to a gene, and the color of the arrows corresponds to the gene’s name.

Notably, the Freshwater biome was enriched for Biological Process (6 GO annotations, *P*-value < 0.01): “Cell motility”, Cellular Component (6 GO annotations, *P*-value < 0.01):” bacterial-type flagellum” and Molecular Function (5 GO annotations, *P*-value < 0.01): “structure of cell wall”. Additionally, more flagellum-related genes were detected in the Freshwater biome than in the other three biomes (Supplementary Fig. S14). All of the evidence supplied by the enrichment sphere model suggests that there were gene vertical accumulation events in the flagellum-related genes (impacting the motility of their host) to respond to the high fluidity of water. Based on the enrichment sphere model, the flagellum-related genes, together with their hosts, were enriched in the Freshwater biome (*P*-value < 0.01, Fig. 2c and Table 1). First, 54 flagellum-related genes (Fig. 4a) were primarily from three phyla: Proteobacteria (1058 counts), Bacteroidetes (783 counts), and Firmicutes (628 counts) (Fig. 4b). All of them were dominant members in the Freshwater biome (relative abundance 28.15%, 15.64%, and 14.25%, respectively), in agreement with previous research^44,45^. Interestingly, a significant positive correlation was identified (*R*^2^ = 0.954, *P*-value < 0.001, Fig. 4c) between the number of flagellum-related genes and the relative abundance of their hosts (Fig. 4d). Due to the high fluidity of the Freshwater biome, species with a better motor ability (the vertical accumulation of flagellum-related genes) may have an advantage in adjusting to environmental stress, as proven by this work (Fig. 4c, d).

Taken together, these two case studies demonstrate the usefulness of the enrichment sphere model in understanding the external factors and internal factors that contribute to the biome–species–function relationship. As a consequence, the enrichment sphere model can help in the construction of general ecological models describing the selection of species, and their genes under different influence factors.

### The enrichment sphere model could guide the identification of biomes

**Fig. 5:**
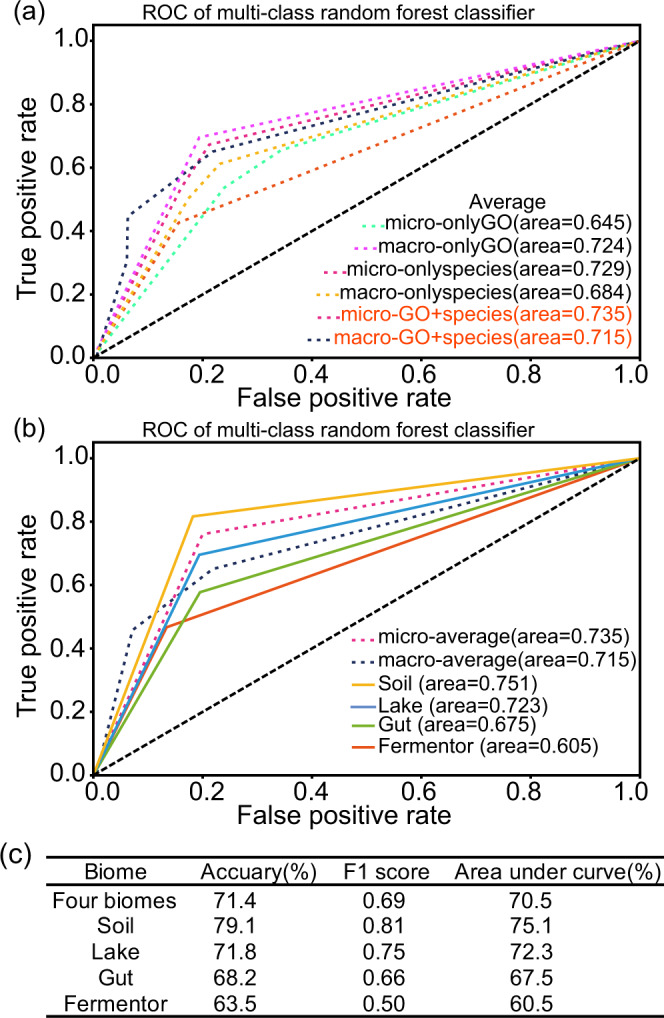
Sample classification results based on the enriched GO annotations and host species information. **a** The ROC analysis of the multiple-classification random forest model. This model was constructed to classify the source biome for metagenome samples, using the enriched GO annotations and host species information and combination of these two datasets (Supplementary Data 2). **b** The ROC analysis of multiple-classification random forest model. First, the classification accuracy of samples from a single biome was evaluated. Second, to evaluate the overall prediction accuracy for the multiple-classification model, the micro-average (obtained by aggregating the contributions of all classes to compute the average metric) and macro-average value (calculated by the metric independently for each class and take the average) were applied. **c** The evaluation results of the classification model used to predict the biome of each sample. The evaluation scores include the accuracy (which indicates the number of right predictions of samples), the F1 score (which indicates the balance of the precision and the recall) and Area Under Curve (measure the ability of a classifier to distinguish between classes).

Since several sets of genes and species have demonstrated enrichment patterns, it is natural to use the enrichment sphere model for guiding the identification of biomes. We validated the biome specificity by developing a classifier that predicts the biome of each sample using the enriched GO annotations of genes and their hosts in the enrichment sphere model (Fig. 5a). Based on the area under the receiver operating characteristic (AUC) curve, the model generated using GO annotations and species distribution performed better than using these two datasets independently. The model accuracy of species+GO annotation was 71.4% across the four biomes (Fig. 5b, c). It is worth noting that in this analysis, we have used 75 GO annotations and 115 species that have shown significant enrichment patterns (Supplementary Data 2, Supplementary Fig. S15), rather than intentionally using biomarkers that were selected with high discrimination power (Supplementary Data 3). For example, in the human gut, whereas the species *Prevotella copri* exhibited strong enrichment patterns in the gut biome (*P*-value 6.23e–10), this species was not identified as a biomarker in the Gut biome (Supplementary Data 3). According to prior research, this species was enriched in gut microbiomes to produce short-chain fatty acids, which are beneficial to human health^47^. However, the moderate accuracy in the identification of biomes has reinforced the notion that these genes and species were enriched. Taken together, by utilizing genes and species which have shown the enrichment patterns, we could accurately identify the microbial community’s habitat biomes. This result exemplified a universal rule governing the biome–species–function relationship, and the model’s moderate accuracy also proved that the diffusion theory of ecology remains viable.

### The 3D structures of proteins could be modeled *de novo* with the guide of the enrichment sphere model

The enrichment sphere approach offers tremendous promise for extracting functional genes from the metagenome, in addition to providing an ecological viewpoint on microbial communities across biomes. One application of this model was to guide the supplementation of homologous sequences for *de novo* protein 3D structure prediction^31,48^. Several copper-resistance and flagellum-related protein families, which were enriched in the Soil and Freshwater biomes (Fig. 2c), remain unsolved in the Pfam database. Based on this enrichment sphere model, these genes’ homologs have been detected with a significantly higher abundance in certain biomes rather than evenly distributed in all the biomes. For example, for Pfam PF12597, there are 183 homologous sequences in the Soil biome, while there are only 125, 39, and 68 in Freshwater, Gut, and Engineered respectively. Therefore, the Soil biome should be selected for supplementing the homologous sequence for Pfam PF12597.

**Fig. 6:**
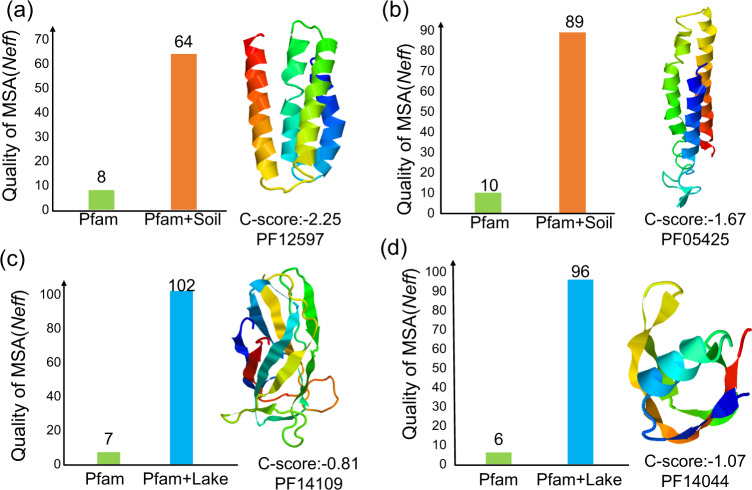
The 3D structure models supplemented by the metagenome from function-enriched biomes. Supplemented with the homologous sequence from metagenome data of the given biome based on the enrichment sphere model, a reliable structure for unsolved protein families was constructed. When supplemented with the metagenome data from the Soil biome (Pfam+Soil), copper resistance proteins PF12597 (**a**) and PF05425 (**b**) had enough homologous sequences to generate reliable 3D structure models. When combined with the metagenome genes from the Freshwater biome (Pfam+Freshwater), the flagellum-related proteins PF14109 (**c**) and PF14044 (**d**) had enough homologous sequences to generate reliable 3D structure models. The quality of multiple sequence alignments was measured by *Neff* scores, where a higher *Neff* score means a higher quality of multiple sequence alignments. A model with C-score over -2.5 indicates a reliable structure. Higher C-scores indicate a more accurate structure.

Based on this approach, the enrichment sphere model could guide the reliable protein 3D structures modeling. For instance, in the Pfam database, the protein structure of copper resistance Pfam PF12597 and PF05425 were unsolved. Based on the enrichment sphere model, their homologous sequences were increased from 182 and 425 in the Pfam database to 365 and 1,022, supplemented by the soil microbiome, respectively. There is enough homologous information for Pfam PF12597 (Fig. 6a) and PF05425 (Fig. 6b) (*Neff* scores 64 and 89, respectively) to model their 3D structures (C-scores -2.25 and -1.67, respectively). Simultaneously, supplemented with homologous sequences from the Freshwater biome, the numbers of homologous sequences for flagellum-related Pfam PF14109 (Fig. 6c) and PF14044 (Fig. 6d) increased, from 285 and 411 in the Pfam database to 689 and 894, respectively. With more homologous information supplemented (*Neff* scores 102 and 96, respectively), their reliable 3D structures could be modeled (C-scores –0.81 and –1.07, respectively).

## Discussion

### Biome ontology information is excellent for examining the dynamic changes of microbial communities in response to external factors

External factors, particularly environmental stresses, exert considerable selection pressure on the microbial community’s structure as characterized by the taxonomical composition and functional profile^49,50^. When confronted with the complex composition of microbial communities, it is crucial to use biological ontological knowledge for dimension reduction and sample clustering^51,52^. We used biome information as biological ontology, and the result confirmed the high value of biome information for dimension reduction and sample clustering: taxonomical composition and functional profiles indicate comparable characteristics within the same biome but uneven distributions among biomes (Fig. 1b and e). Therefore, biome information can assist us in shifting our focus from determining the differences among thousands of samples to determining the difference between selected biomes. In this way, biome information would help reveal the important role of environmental stresses on this biome-specific species composition and functional profile. This is a central topic due to its relevance to basic mechanisms of eco-evolutionary and applied questions^53,54^.

### The formation of the enrichment patterns from the ecological perspective

We observed that both external factors and internal factors have impacted the biome–species–function relationship, and found that different biomes have presented different kinds of environmental stresses, resulting in the survival advantage of certain genes in response to this stress^55,56^. As a result, these genes, along with their hosts, were enriched in their living biome rather than evenly distributed in all biomes. Unscrambling this relationship has spawned the emergence of the enrichment sphere model (Fig. 2c). Two case studies (Figs. 3 and 4) illuminate how external and internal factors contribute to the enrichment of these genes and their hosts, in their respective biomes. The horizontal transfer events across species (Fig. 3) and vertical accumulation in single species (Fig. 4) of enriched genes confer resistance to environmental stresses. The enriched genes and their hosts would accelerate their advancement in the microbial community^19,57^. The horizontal transfer of copper resistance genes across species **(**Fig. 3c, d**)** may help their host to cope with soil-specific copper pollution^58,59^. Concurrently, vertical accumulation of the genes within single species would reflect its influence on the enrichment patterns: in the Freshwater biome with higher fluidity, more flagellum-related genes would benefit their hosts by conferring greater mobility. Reflected by our statistical analysis, species with a greater abundance in the Freshwater biome have more flagellum-related genes (Fig. 4c).

In conclusion, the enriched genes would help their host adapt to the environmental stress from external factors that contribute to their host’s enrichment in their biomes. Additionally, both horizontal gene transfer and vertical gene accumulation have sped up the enrichment of environmental stress-resistance genes and their hosts. This is a win-win strategy for genes and their hosts. The enrichment sphere model clarifies the underlying processes to influence the biome–species–function relationship.

### The formation of the “spheres” for the enriched genes: gradient of complexity

While external factors (environmental stresses) are biome-specific, their gradient of intrinsic complexity cannot be overlooked when analyzing their influence on the microbial community. The intrinsic complexity of biomes exists in the ontologically adjacent biomes, which share a similar set of environmental factors but have different parameters^50^. For example, the temperature has a great effect on the Soil biome^47^, and fluctuations in temperature within the Soil biome would likewise influence the composition of the microbial community. Hence, from an evolutionary perspective, to cope with this intrinsic complexity of environmental stresses, a sufficient number of genetic mutations need to be accumulated rather than simple gene duplication^60^, demonstrating the functional redundancy and structural resilience of the microbial community^61,62^. As shown in our enrichment sphere model, this would imply that similar functions would be enriched within the ontologically adjacent biomes, resulting in the observed “spheres” of GO annotation. Additionally, this “redundancy” is critical for environmental stress adaptation, which has been proved in previous research^63,64^. Under the influence of long-term selection on these genetic mutations, the genes with different functions would be selected to cope with these intrinsic complexities of environmental stresses. The redundancy and robustness of the microbial community have been partially reflected in prior studies on microbiome alterations during long-term travel^10,11^. Diet and habit would significantly disrupt the gut microbiota during travel. The gut microbiome would adjust to this perturbation by raising the abundance of the microbe and the functional genes that deal with them^11^ and by extending the abundance of associated genes via horizontal gene transfer^10^. After returning to Beijing, the gut microbiome’s taxonomic profile and functional composition would revert to its initial state (Supplementary Figure S16).

Moreover, we emphasized that the enrichment sphere model could guide the classification of samples in different biomes. On one hand, this has exemplified a universal rule governing the biome–species–function relationship. And on the other hand, the model’s moderate accuracy proved that the diffusion theory of ecology remains valid. We hoped that our enrichment sphere model and the in-depth examination of this model may contribute to the later discovery of the ecological and evolutionary mechanisms of microbial communities.

In addition, we noted that the enrichment sphere model could be utilized to facilitate the mining of functional genes. The protein 3D structure modeling results demonstrate that mutations of genes in response to environmental stress have been extensively accumulated (Fig. 5). To model the 3D structures of proteins, a sufficient number of genetic mutations should be accumulated^65,66^. Hence, the successful prediction of the protein 3D structures (e.g., copper resistance Pfams PF12597 and PF05425; flagellum-related Pfams PF14109 and PF14044) has illustrated the accumulation of gene mutations, which was potentially caused by the dynamic changes in environmental conditions.

In summary, an enrichment sphere model that can reflect the biome–species–function relationships have been established in this study, and the model has demonstrated the enrichment of both species and functions in specific biomes. These results have profound implications in ecology and evolution: the external factors (environmental stresses with a gradient of complexity) and internal factors (vertical gene accumulation and horizontal gene transfer) have shaped the species and functional genes to the current state, i.e., they are enriched rather than being dispersed. This research elucidated the biogeography of microbial genes and the adaptive development of microbial communities. The enrichment sphere model might, for instance, determine the enriched biome for genes with specific functions, infer the enriched biome of homologous sequences for proteins without known structure, and compute the reaction mechanism of a microbial community to an environmental change. It has contributed to the promotion of small molecule drug mining, the 3D structural modeling of unresolved proteins, and the prediction of a microbial community’s development path.

These findings revealed that the enrichment model exposes the ecological and evolutionary implications of microbial communities in their living environment. However, it is worth noting that, due to the nature of sample accessibility and sequencing technology, we would encounter an uneven distribution of samples from various biomes, which might influence the statistical results. Consequently, greater effort should be devoted to proving important findings in future research.

We realized that we have merely uncovered the tip of the iceberg regarding the biome–species–function relationship, a relationship to date that has been poorly understood, with many puzzles remaining unsolved: How can the evolution of the genes and species be linked with the evolution of the community? How many of the niche-specific functional genes are also mobile elements in the community? How are the dynamic patterns of the niche-specific species and genes formed when the community is under stress? All of these questions need to be addressed in subsequent studies.

## Methods

### Microbial community cohorts collected from four representative biomes

We collected metagenome data from the European Bioinformatics Institute (EBI) database, which is an organized database according to the habitat environments (biomes)^9^. The first layer of this database is divided into three biomes: “Engineered”, “Environmental” and “Host-associated”^67^. To cover the representative biomes on Earth^31,68^, Samples in MGnify (https://www.ebi.ac.uk/metagenomics/) were downloaded, filtered by biomes (Engineered, Gut, Freshwater, and Soil), Experiment type:”metagenome” and release date: later than January 2019. Finally, 1,705 microbial samples were obtained. Among them, the biome “Engineered” was selected as a representative biome for the “Engineered” biome; the biomes “Soil” and “Freshwater” were selected as representative biomes for the “Environmental” biome; the “Gut” biome that includes human and animal (mice, pigs, cattle) intestines were selected for the “Host-associated” biome. Supplementary Data 4 provided detailed information for these samples.

### Analysis of taxonomical profile and functional composition from four representative biomes

After all the raw reads of 1705 samples were downloaded, the FastQC (version 0.11.9, https://www.bioinformatics.babraham.ac.uk/projects/fastqc/) was used to filter out low-quality reads, then a *de novo* assembler MEGAHIT v1.0^69^ was used to assemble these reads into contigs. Reads in different datasets were assembled individually. Option–meta-large was used for assembling. Contigs that were shorter than 500 nucleotides were discarded.

To profile the taxonomical composition of microbial communities, MetaPhlAn 2.0^70^ was used with default settings. To adjust the batch effects among different studies, the R package MMUPHin (version 1.10.3)^71^ was used, based on the relative abundance of species from 1,705 samples obtained from MetaPhlAn 2.0.

For functional annotation, Prodigal (version 2.6) was used to recognize open reading frames (ORFs) in assembled contigs in each sample^72^. Options -c and -m were added to the command line to prevent genes from running off edges and avoid building genes across runs of N. ORFs that are shorter than 150 nucleotides were discarded. CD-HIT v4.6 was used to cluster identical ORFs in each study^73^. The identity threshold for sequence clustering was set to 95%, and the alignment must cover at least 90% of the shorter sequence. Local sequence identity was used and both + /+ and + /- alignments were performed. Clustering was performed by using CD-HIT’s default algorithm. In each cluster, only the representative sequence marked by CD-HIT was kept. Two sequences that met the clustering threshold but were from different datasets were not considered redundant. Predicted non-redundant nucleotide sequences in each dataset were translated into amino acid sequences using prodigal. Based on the ORFs, the protein domain distribution was searched against Conserved Domain Database (CDD) database (https://www.ncbi.nlm.nih.gov/cdd/) using Blastx at the local server, which returns the gene information for these predicted non-redundant nucleotide sequences. Blastx searching was performed with an *e*-value threshold of 1e–10. A query sequence was annotated as a conserved domain if the first high-score pair (HSP) of its top hit showed a percent identity ≥60% and a query coverage ≥70% in CDD. The number of conserved domains detected in each study was normalized based on single-copy genes. Based on the gene information returns from the CDD database, the gene was annotated into GO based on the R package biomart (version 2.54.0, https://bioconductor.org/packages/release/bioc/html/biomaRt.html). The proportions of each GO annotation in the four biomes were calculated. To calculate the relative abundance (unit: per million reads) of each Go annotation in a sample, Bowtie2 (version 2.4.3, https://bowtie-bio.sourceforge.net/bowtie2/index.shtml) was applied to match the genes to all the raw reads, so determining the proportion of reads of the genes identified under the GO in the total number of reads. Further normalization of the metagenomic functional gene was performed by the Trimmed mean of *M*-values based on the edgeR package (version 3.40.2, https://bioconductor.org/packages/release/bioc/html/edgeR.html). To estimate the accuracy of the GO annotation process, 100 bacterial proteins (filtered by taxonomy_id: 2) were randomly chosen from the UniProtKB database (https://www.uniprot.org/uniprotkb?query = *), downloading the protein sequence and related GO annotations. After annotating in our annotation process, the result is regarded as correct if it corresponds to the functional result of the annotation in the Uniport database. In the end, the accuracy is 100%. To adjust the batch effects among different studies, the R package MMUPHin (version 1.10.3) was used, based on the relative abundance of functional composition from 1,705 samples based on GO annotation. R package pheatmap (version 1.0.12) was applied to illustrate the functional composition, with the sample cluster method set as the euclidean metric.

### Differential characterization of metagenome data from four biomes based on their taxonomical composition and functional profile

To investigate the biome–species–function relationship (Supplementary Fig. S17), the taxonomical composition and functional profiles across biomes were first examined. The alpha diversity of samples from four biomes was examined by the Shannon index and Simpson index by R package phyloseq (version 1.34.0). For relative abundance composition adjusted by MMUPHin, PCoA analysis was conducted using the species with relative abundance >0.1%, based on the Canberra distance. PCoA analysis was also used to be detected the differences in protein domain and GO annotations across biomes, based on the Bray-Curtis distance.

### Exploration of the enrichment sphere model

Since the Hypergeometric tests were much more suitable for the count data. Adjusted by batch effect, the relative abundance proportions of functional compositions were multiplied by reads counts and rounded to the nearest integer before being tested. And the enrichment sphere model was constructed in the following manner:

(1). We evaluated if a function domain processed a significantly different distribution in one biome than in others. To do this, univariate hypergeometric tests were performed using Scipy package (http://www.scipy.org/) on each species against each biome.

(2). We evaluate if the species and GO annotations (a further integration of the function domain) significantly varied across the four biomes. For species and GO annotations in four biomes, univariate hypergeometric tests were performed on each species or GO annotations against each biome.

(3). Based on the enriched GO annotations identified in step (2), further enrichment analysis was performed: using a multivariate hypergeometric test, their neighboring GO annotations (ontologically adjacent or parent in the GO ontology with similar functions) were also tested to check if they are enriched in the same biome. This test began with a single neighboring GO annotation for enriched GO annotations. Then, we included more neighbor GO annotations gradually until the *P*-value exceeded the threshold (0.01). These GO annotations would form a function sphere in a specific biome. The univariate hypergeometric test was also conducted to identify if their hosts were enriched in the same biomes. This test was performed using the R package BiasedUrn (version 1.07, https://cran.r-project.org/web/packages/BiasedUrn).

(4). These biome, species, and function relationships based on the enrichment analysis were integrated by an enrichment sphere model, based on the GO ontology annotation and the function sphere calculated in the preceding steps.

As a benchmark of the enrichment result, all the enriched functions were tested by LEfSe analysis (https://huttenhower.sph.harvard.edu/lefse/), using relative abundance distribution for four biomes. Only the function with LDA value > 4 and *P*-value < 0.5 were selected for further analysis.

### The comparative physiological and evolutionary investigations reveal the species–function relationship based on the enrichment sphere model

To investigate the biome–species–function relationship (Supplementary Fig. S17) mirrored by the enrichment sphere model, comparative physiological and evolutionary analyses were performed: To construct the phylogenetic tree, the species were classified the NCBI taxonomy database^74^. PhyloT (http://phylot.biobyte.de/) was used to map the species to NCBI common tree (https://www.ncbi.nlm.nih.gov/Taxonomy/CommonTree/wwwcmt.cgi); subsequently, the results were visualized and modified by the online tools iTOL^75^. To detect gene communications in the microbial community, possible horizontal gene transfer events were identified using a reference-independent tool MetaCHIP^27^ (version 1.10.0) with default parameters. In MetaCHIP, the detected genes were limited within the enriched GO annotations detected in the enrichment sphere model. To illustrate the genetic structure of the genome, arrow maps were drawn using the R package gggenes (version 0.4.1, https://cran.r-project.org/web/packages/gggenes).

To construct the phylogenetic tree of species, PhyloT (http://phylot.biobyte.de/) was used to map the taxonomy IDs to the NCBI common tree (https://www.ncbi.nlm.nih.gov/Taxonomy/CommonTree/wwwcmt.cgi), and subsequently, the results were visualized and modified by an online tool iTOL (http://itol.embl.de/itol.cgi).

### The prediction model based on genes and species that show enrichment patterns

For building a prediction model, we have used 75 GO annotations and 115 species that have shown significant enrichment patterns (Supplementary Data 2). For classification, a random forest classifier, as implemented in scikit-learn (https://scikit-learn.org/) with 100 trees. The stability selection, which controls for biome-selection error rate, was performed by R package stabs (version 0.6-4, https://cran.r-project.org/web/packages/stabs/). Tenfold, stratified cross-validation was used to evaluate the classification accuracy. The parameters were determined using grid search to gain the best accuracy of the constructed model.

### Statistics and reproducibility

After downloading all of the raw data from 1,705 samples, the FastQC (version 0.11.9, http://www.bioinformatics.babraham.ac.uk/projects/fastqc/) was used to filter away low-quality reads, resulting in 68.51 billion high-quality reads. On the basis of these reads, the genome of the species was assembled using the default parameters of the reference-independent tool MetaCHIP (version 1.10.0)^27^.

Using Prodigal (version 2.6) for functional composition and enrichment analysis, 4.25 billion genes were predicted (Soil: 1.31 billion, Freshwater: 0.74 billion, Gut: 2.13 billion, and Engineered: 0.13 billion). The Gene Ontology (GO) database was used to further integrate the functional genes, and the four biomes were identified based on the number of GO annotations present in each (Soil: 520 million, Freshwater: 120 million, Gut: 1530 million, and Engineered: 67 million). Corrected for batch effect, relative abundance proportions of GO annotation were multiplied by counts of reads and adjusted to the nearest integer before to enrichment analysis testing. Using the R package BiasedUrn (version 1.07, https://cran.r-project.org/web/packages/BiasedUrn), we performed univariate hypergeometric tests on the enrichment analysis of GO annotations in distinct biomes. On the basis of Bray-Curtis distance, PCoA was also utilized to uncover changes in protein domain and GO annotations among biomes.

As a consequence, 75 GO annotations and 115 species with significant enrichment patterns were found.

### The *de novo* proteins 3D structure modeling supplemented by the metagenome data

Pfam (version 32, widely used version) is a database containing 17,929 protein families that are clustered by protein function or sequence similarity^76^. To model the structure of the Pfam families, the metagenome data from habitat biomes was employed to supplement the Pfam homologous sequences using DeepMSA^77^. Based on the collected multiple sequence alignments, residue-residue contact maps were constructed using five deep-learning and co-evolution-based predictors, TripletRes^78^, ResTriplet^79^, NeBcon^80^, ResPRE^81^, and ResPLM^82^. The protein 3D structures were predicted based on the residue-residue contact maps by C-I-TASSER^83^. The accuracy of the 3D structure model for each protein was estimated by the TM-score^84^ and C-score^83^.

### Reporting summary

Further information on research design is available in the Nature Portfolio Reporting Summary linked to this article.

## Supplementary information

Supplementary Information

Description of Additional Supplementary Files

Supplementary Data 1

Supplementary Data 2

Supplementary Data 3

Supplementary Data 4

Reporting Summary

## Data Availability

The data that support the findings of this study are all openly available. Supplementary Data 4 contains the accession numbers for all the metagenomes used. The intermediate files were available at https://github.com/HUST-NingKang-Lab/biome-species-function-relationship.

## Code availability

The codes used in our research were available at https://github.com/HUST-NingKang-Lab/biome-species-function-relationship.

## References

[1] Forslund K (2015). Disentangling type 2 diabetes and metformin treatment signatures in the human gut microbiota. Nature.

[2] Thompson LR (2017). A communal catalogue reveals Earth’s multiscale microbial diversity. Nature.

[3] Kadosh E (2020). The gut microbiome switches mutant p53 from tumour-suppressive to oncogenic. Nature.

[4] Munita J. M. & Arias C. A. Mechanisms of antibiotic resistance. *Microbiol. Spectr.*10.1128/microbiolspec.VMBF-0016-2015 (2016). 10.1128/microbiolspec.VMBF-0016-2015 PMC4888801 27227291

[5] Zilber-Rosenberg I, Rosenberg E (2021). Microbial driven genetic variation in holobionts. FEMS Microbiol. Rev..

[6] Mallott EK, Amato KR (2021). Host specificity of the gut microbiome. Nat. Rev. Microbiol..

[7] Akbar S (2022). Understanding host-microbiome-environment interactions: Insights from Daphnia as a model organism. Sci. Total Environ..

[8] Gacesa R (2022). Environmental factors shaping the gut microbiome in a Dutch population. Nature.

[9] Mitchell AL (2020). MGnify: the microbiome analysis resource in 2020. Nucleic Acids Res..

[10] Bengtsson-Palme J (2015). The human gut microbiome as a transporter of antibiotic resistance genes between continents. Antimicrob. Agents Chemother..

[11] Liu H (2019). Resilience of human gut microbial communities for the long stay with multiple dietary shifts. Gut.

[12] Jackrel SL, Yang JW, Schmidt KC, Denef VJ (2021). Host specificity of microbiome assembly and its fitness effects in phytoplankton. ISME J..

[13] Lloyd-Price J (2017). Strains, functions and dynamics in the expanded human microbiome project. Nature.

[14] Wu WK (2020). Characterization of TMAO productivity from carnitine challenge facilitates personalized nutrition and microbiome signatures discovery. Microbiome.

[15] Navarro-Munoz JC (2020). A computational framework to explore large-scale biosynthetic diversity. Nat. Chem. Biol..

[16] Sberro H (2019). Large-scale analyses of human microbiomes reveal thousands of small, novel genes. Cell.

[17] Pereira GV (2021). Degradation of complex arabinoxylans by human colonic bacteroidetes. Nat. Commun..

[18] Wang C (2021). Organic matter stabilized Fe in drinking water treatment residue with implications for environmental remediation. Water Res..

[19] Le Roux F, Blokesch M (2018). Eco-evolutionary dynamics linked to horizontal gene transfer in vibrios. Annu. Rev. Microbiol.

[20] Oladeinde A (2021). Horizontal gene transfer is the main driver of antimicrobial resistance in Broiler chicks infected with Salmonella enterica serovar Heidelberg. mSystems.

[21] Arnold BJ, Huang IT, Hanage WP (2021). Horizontal gene transfer and adaptive evolution in bacteria. Nat. Rev. Microbiol..

[22] Rissanen AJ (2021). Vertical stratification patterns of methanotrophs and their genetic controllers in water columns of oxygen-stratified boreal lakes. FEMS Microbiol. Ecol..

[23] Zhang R (2021). Winner-takes-all resource competition redirects cascading cell fate transitions. Nat. Commun..

[24] Huus KE (2021). Cross-feeding between intestinal pathobionts promotes their overgrowth during undernutrition. Nat. Commun..

[25] Yazdankhah S, Skjerve E, Wasteson Y (2018). Antimicrobial resistance due to the content of potentially toxic metals in soil and fertilizing products. Micro. Ecol. Health Dis..

[26] Brito IL (2021). Examining horizontal gene transfer in microbial communities. Nat. Rev. Microbiol..

[27] Song W, Wemheuer B, Zhang S, Steensen K, Thomas T (2019). MetaCHIP: community-level horizontal gene transfer identification through the combination of best-match and phylogenetic approaches. Microbiome.

[28] Durrant MG, Li MM, Siranosian BA, Montgomery SB, Bhatt AS (2020). A bioinformatic analysis of integrative mobile genetic elements highlights their role in bacterial adaptation. Cell Host Microbe.

[29] Tokeshi M. Species abundance patterns and community structure. *Adv. Ecol. Res.* **24**, 111–186 (1993).

[30] Hou J (2020). Microbial succession during the transition from active to inactive stages of deep-sea hydrothermal vent sulfide chimneys. Microbiome.

[31] Wang Y (2019). Fueling ab initio folding with marine metagenomics enables structure and function predictions of new protein families. Genome Biol..

[32] Mitchell AL (2019). MGnify: the microbiome analysis resource in 2020. Nucleic Acids Res..

[33] Coelho LP (2021). Towards the biogeography of prokaryotic genes. Nature.

[34] Panwar P (2020). Influence of the polar light cycle on seasonal dynamics of an Antarctic lake microbial community. Microbiome.

[35] Piwosz K (2020). Light and primary production shape bacterial activity and community composition of aerobic anoxygenic phototrophic bacteria in a microcosm experiment. mSphere.

[36] Kummen M (2021). Altered gut microbial metabolism of essential nutrients in primary sclerosing cholangitis. Gastroenterology.

[37] Then CK, Paillas S, Wang X, Hampson A, Kiltie AE (2020). Association of Bacteroides acidifaciens relative abundance with high-fibre diet-associated radiosensitisation. BMC Biol..

[38] Luo L (2021). Comparison of bacterial communities and antibiotic resistance genes in oxidation ditches and membrane bioreactors. Sci. Rep..

[39] Gene Ontology C. (2021). The gene ontology resource: enriching a GOld mine. Nucleic Acids Res..

[40] Rogiers T (2021). Soil microbial community structure and functionality changes in response to long-term metal and radionuclide pollution. Environ. Microbiol..

[41] Zhang J (2021). Distinction between Cr and other heavy-metal-resistant bacteria involved in C/N cycling in contaminated soils of copper producing sites. J. Hazard Mater..

[42] Jabbarzadeh M, Fu HC (2020). Large deformations of the hook affect free-swimming singly flagellated bacteria during flick motility. Phys. Rev. E.

[43] Johnson S (2021). Molecular structure of the intact bacterial flagellar basal body. Nat. Microbiol..

[44] Ji B, Liang J, Ma Y, Zhu L, Liu Y (2019). Bacterial community and eutrophic index analysis of the East Lake. Environ. Pollut..

[45] Zhang L, Zhao T, Shen T, Gao G (2019). Seasonal and spatial variation in the sediment bacterial community and diversity of Lake Bosten, China. J. Basic Microbiol..

[46] Song J (2020). A converging subset of soil bacterial taxa is permissive to the IncP-1 plasmid pKJK5 across a range of soil copper contamination. FEMS Microbiol Ecol..

[47] Asnicar F (2021). Microbiome connections with host metabolism and habitual diet from 1,098 deeply phenotyped individuals. Nat. Med..

[48] Yang P, Zheng W, Ning K, Zhang Y (2021). Decoding the link of microbiome niches with homologous sequences enables accurately targeted protein structure prediction. Proc. Natl Acad. Sci. USA.

[49] After the Integrative Human Microbiome Project, what’s next for the microbiome community? *Nature* **569**, 599 (2019). 10.1038/d41586-019-01674-w 31142868

[50] Alberdi A, Andersen SB, Limborg MT, Dunn RR, Gilbert MTP (2021). Disentangling host-microbiota complexity through hologenomics. Nat. Rev. Genet..

[51] Huss J (2014). Methodology and ontology in microbiome research. Biol. Theory.

[52] Vangay P (2021). Microbiome metadata standards: report of the national microbiome data collaborative’s workshop and follow-on activities. mSystems.

[53] Cheng YT, Zhang L, He SY (2019). Plant-microbe interactions facing environmental challenge. Cell Host Microbe.

[54] Kurilshikov A (2021). Large-scale association analyses identify host factors influencing human gut microbiome composition. Nat. Genet..

[55] Avila-Magana V (2021). Elucidating gene expression adaptation of phylogenetically divergent coral holobionts under heat stress. Nat. Commun..

[56] Teles F, Wang Y, Hajishengallis G, Hasturk H, Marchesan JT (2021). Impact of systemic factors in shaping the periodontal microbiome. Periodontol. 2000.

[57] Zhu B (2016). Multi-omics analysis of niche specificity provides new insights into ecological adaptation in bacteria. ISME J..

[58] Ghobadi R (2021). Enhanced copper removal from contaminated kaolinite soil by electrokinetic process using compost reactive filter media. J. Hazard Mater..

[59] Miao C, Yao SS, Liu SJ, Zhang K (2021). Effect of water-soluble thiourea formaldehyde (WTF) on soil contaminated with high copper () concentration. J. Hazard Mater..

[60] Garrett-Bakelman FE (2019). The NASA Twins Study: a multidimensional analysis of a year-long human spaceflight. Science.

[61] De Anda V (2018). Understanding the mechanisms behind the response to environmental perturbation in microbial mats: a metagenomic-network based approach. Front. Microbiol..

[62] Eng A, Borenstein E (2018). Taxa-function robustness in microbial communities. Microbiome.

[63] Ananbeh H (2020). Soil protein as a potential antimicrobial agent against methicillin -resistant Staphylococcus aureus. Environ. Res..

[64] Cao Y (2020). Copper stress in flooded soil: Impact on enzyme activities, microbial community composition and diversity in the rhizosphere of Salix integra. Sci. Total Environ..

[65] Eisenstein M (2021). Artificial intelligence powers protein-folding predictions. Nature.

[66] Hameduh T, Haddad Y, Adam V, Heger Z (2020). Homology modeling in the time of collective and artificial intelligence. Comput Struct. Biotechnol. J..

[67] Mitchell AL (2018). EBI Metagenomics in 2017: enriching the analysis of microbial communities, from sequence reads to assemblies. Nucleic Acids Res..

[68] Ovchinnikov S (2017). Protein structure determination using metagenome sequence data. Science.

[69] Li D, Liu CM, Luo R, Sadakane K, Lam TW (2015). MEGAHIT: an ultra-fast single-node solution for large and complex metagenomics assembly via succinct de Bruijn graph. Bioinformatics.

[70] Truong DT (2015). MetaPhlAn2 for enhanced metagenomic taxonomic profiling. Nat. Methods.

[71] Ma S (2022). Population structure discovery in meta-analyzed microbial communities and inflammatory bowel disease using MMUPHin. Genome Biol..

[72] Hyatt D (2010). Prodigal: prokaryotic gene recognition and translation initiation site identification. BMC Bioinforma..

[73] Fu L, Niu B, Zhu Z, Wu S, Li W (2012). CD-HIT: accelerated for clustering the next-generation sequencing data. Bioinformatics.

[74] Federhen S (2012). The NCBI taxonomy database. Nucleic Acids Res..

[75] Letunic I, Bork P (2016). Interactive tree of life (iTOL) v3: an online tool for the display and annotation of phylogenetic and other trees. Nucleic acids Res..

[76] Mistry J (2021). Pfam: The protein families database in 2021. Nucleic Acids Res..

[77] Zhang C, Zheng W, Mortuza SM, Li Y, Zhang Y (2020). DeepMSA: constructing deep multiple sequence alignment to improve contact prediction and fold-recognition for distant-homology proteins. Bioinformatics.

[78] Li Y (2021). Deducing high-accuracy protein contact-maps from a triplet of coevolutionary matrices through deep residual convolutional networks. PLoS Comput. Biol..

[79] Li Y, Zhang C, Bell EW, Yu DJ, Zhang Y (2019). Ensembling multiple raw coevolutionary features with deep residual neural networks for contact-map prediction in CASP13. Proteins.

[80] He B, Mortuza SM, Wang Y, Shen HB, Zhang Y (2017). NeBcon: protein contact map prediction using neural network training coupled with naive Bayes classifiers. Bioinformatics.

[81] Yilmaz B (2019). Microbial network disturbances in relapsing refractory Crohn’s disease. Nat. Med..

[82] Zheng W (2019). Deep-learning contact-map guided protein structure prediction in CASP13. Proteins.

[83] Zheng W (2021). Folding non-homologous proteins by coupling deep-learning contact maps with I-TASSER assembly simulations. Cell. Rep. Methods.

[84] Zhang Y, Skolnick J (2004). Scoring function for automated assessment of protein structure template quality. Proteins.

