# Supplementary material for: Microbiome-based enrichment pattern mining has enabled a deeper understanding of the biome–species–function relationship
Source: Commun Biol. 2023 Apr 10;6:391. doi: 10.1038/s42003-023-04753-x (PMC10085995; doi:10.1038/s42003-023-04753-x)
Supplement: Supplementary file 1 — Supplementary Information [file 42003_2023_4753_MOESM1_ESM.pdf]

Supplementary file for "Microbiome-based enrichment pattern mining has enabled deeper understanding of the biome–species–function relationship"

Pengshuo Yang[1,2#], Xue Zhu[1,#], Kang Ning[1,2*]

[1]Key Laboratory of Molecular Biophysics of the Ministry of Education, Hubei Key Laboratory of Bioinformatics and Molecular-imaging, Center of AI Biology, Department of Bioinformatics and Systems Biology, College of Life Science and Technology, Huazhong University of Science and Technology, Wuhan 430074, China

[2]Institute of Medical Genomics, Biomedical Sciences College, Shandong First Medical University, Shandong 250117, China

[#] These two authors contributed equally to this work.

*Correspondence should be addressed to K.N (Email: ningkang@hust.edu.cn)

Content

Supplementary Figures......3

Figure S1. Samples statistic results for the four biomes. 3

Figure S2. species distribution on phylum level for samples in the four biomes. 4

Figure S3. Alpha diversity for four biomes.......5

Figure S4. Protein distribution for four biomes. 6

Figure S5. Share and specific GO annotations for four biomes.......7

Figure S6. Functional profile of four biomes based on the HUMAnN 2.0. 8

Figure S7. The function enrichment in four biomes based on the GO annotation.......9

Figure S8. GO distributions for the Soil biome. 10

Figure S9. GO distributions for the Freshwater biome. 11

Figure S10. GO distributions for the Gut biome.......12

Figure S11. GO distributions for the Engineered biome. 13

Figure S12. Correlation between the number of Copper genes and the prevalence of the host species in Soil biome.......14

Figure S13. Phylogenetic tree constructed by copper gene family(left) and whole genome (right). 15

Figure S14. Statistic result of flagellum genes between four biomes. 16

Figure S15. Feature selection process for random forest model. 17

Figure S16. PCoA results based on the functional annotations from previous work (Liu

H, *et.al.* Gut. 2019)..18
Figure S17. Workflow for the examination of the "biome-species-function" relationship.
....19

Supplementary Figures

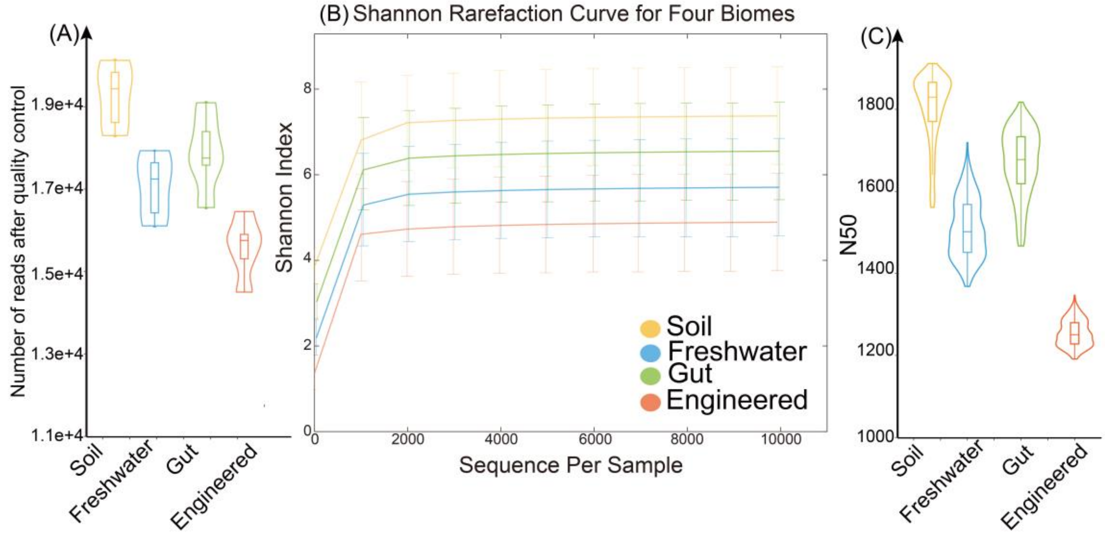

Figure S1. Samples statistic results for the four biomes. (A) Number of reads after quality control. Processed by FastQC, the reads with low quality were removed. **(B) Shannon Rarefaction Curve for Four Biomes.** This curve indicates that our samples could cover all the representative species. **(C) N50 result for contig assemble.** This result reflects that we have assembled with long-read contigs.

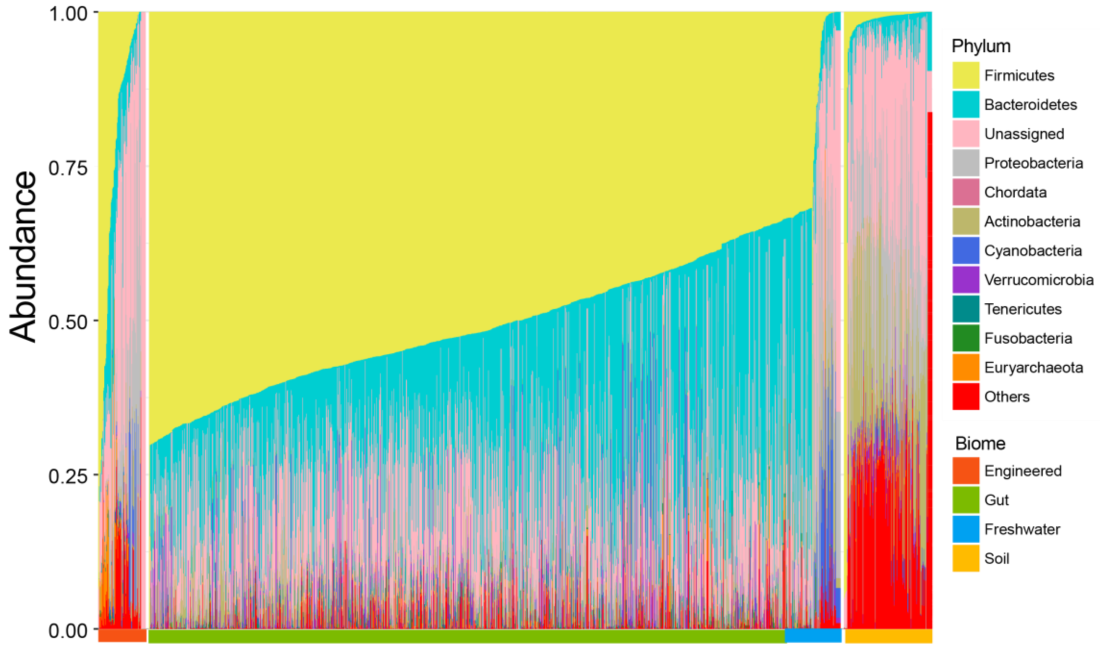

Figure S2. species distribution on phylum level for samples in the four biomes. The species distribution is divided based on the collected biomes and labeled with different colors. Calculated by the average count in all samples, the top 10 phyla are illustrated and ranked. Unassigned means the species cannot be identified by a known phylum. Others represent the combination of rest phyla.

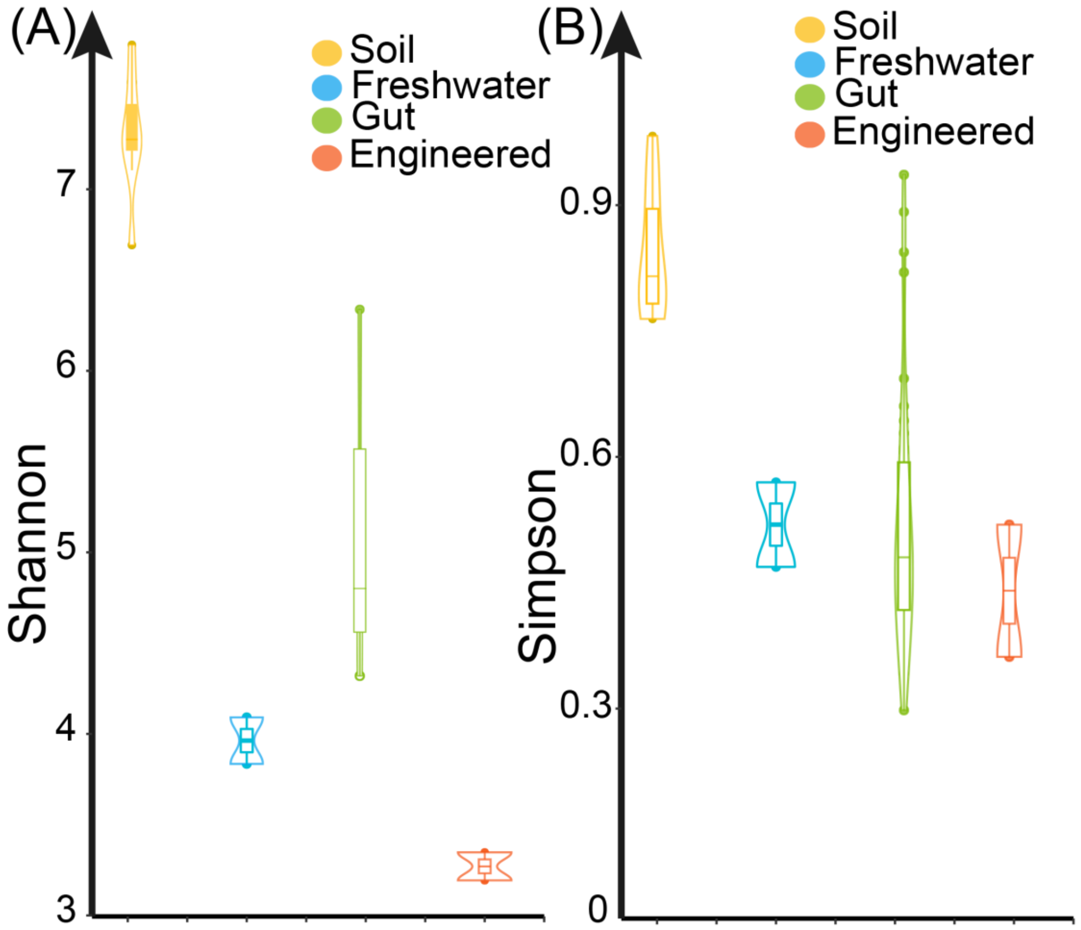

Figure S3. Alpha diversity for four biomes. (A) The species diversity for four biomes is measured by the Shannon index. For four biomes, the average Shannon index was 7.64 (Soil), 3.85 (Freshwater), 4.28 (Gut) and 3.16 (Engineered), respectively. **(B)** The species diversity for four biomes is measured by the Simposon index. For four biomes, the average Simpson index was 0.85 (Soil), 0.56 (Freshwater), 0.57 (Gut) and 0.42 (Engineered), respectively.

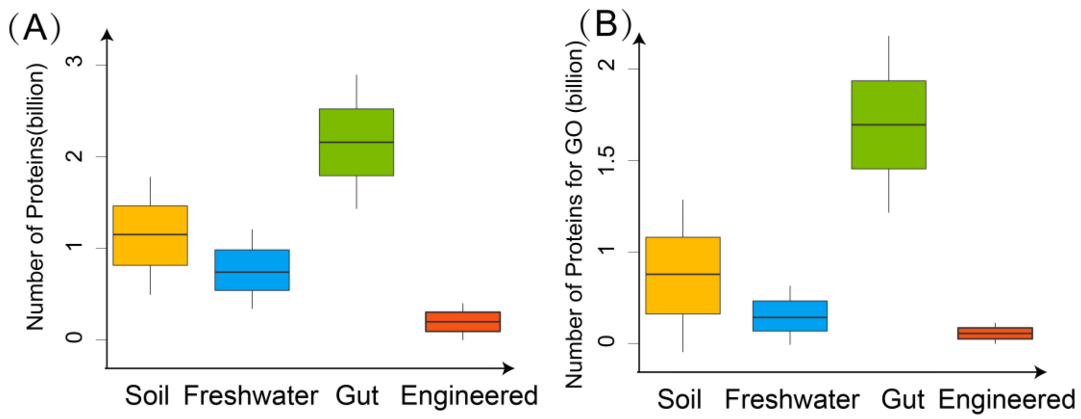

Figure S4. Protein distribution for four biomes. (A)The number of predicted proteins for four biomes. Based on the protein prediction and non-redundancy process (100% identity), the number of proteins was calculated for four biomes. (B)After being annotated by the GO database, the number of aligned proteins was listed for four biomes.

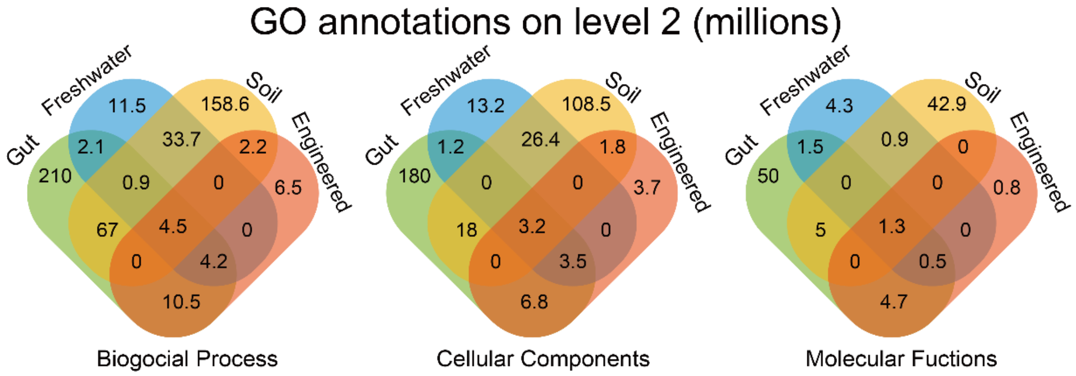

Figure S5. Share and specific GO annotations for four biomes. The common and unique functional distributions for the four biomes. The number labeled in the figure means the number (in millions) of specific or sheared genes annotated by the GO database on gene ontology (level 2), for Biological Process, Celluar Compositions and Molecular Functions.

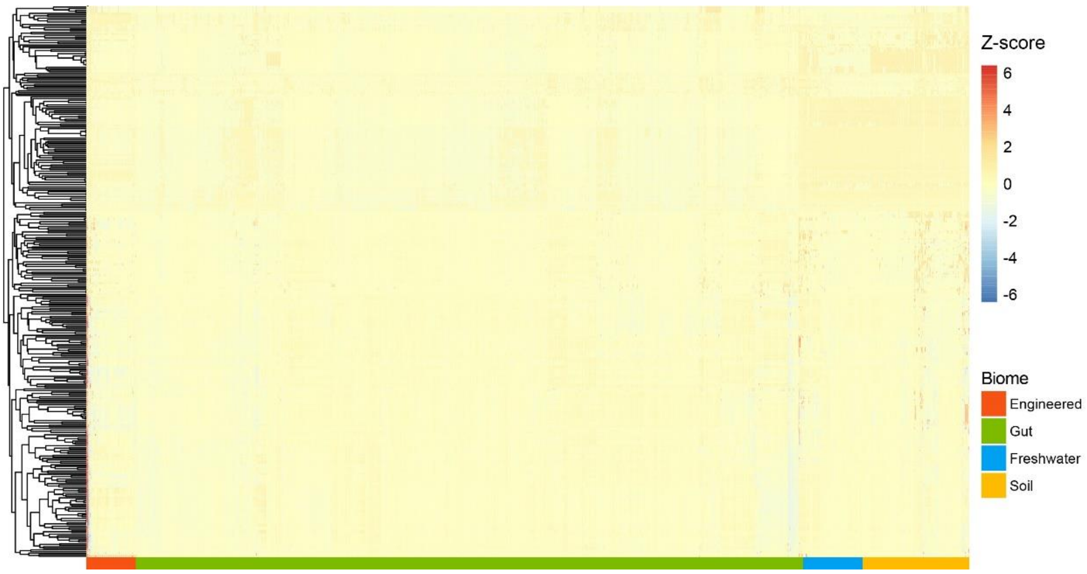

Figure S6. Functional profile of four biomes based on the HUMAnN 2.0. In the heatmap, each row means a function annotation identified by HUMAnN 2.0 (https://huttenhower.sph.harvard.edu/humann2/). Each column means a sample, labeled with its source biome. The function annotation was clustered based on Euclidean Distance. The biome-specific clusters would also be observed based on HUMAnN 2.0 annotation.

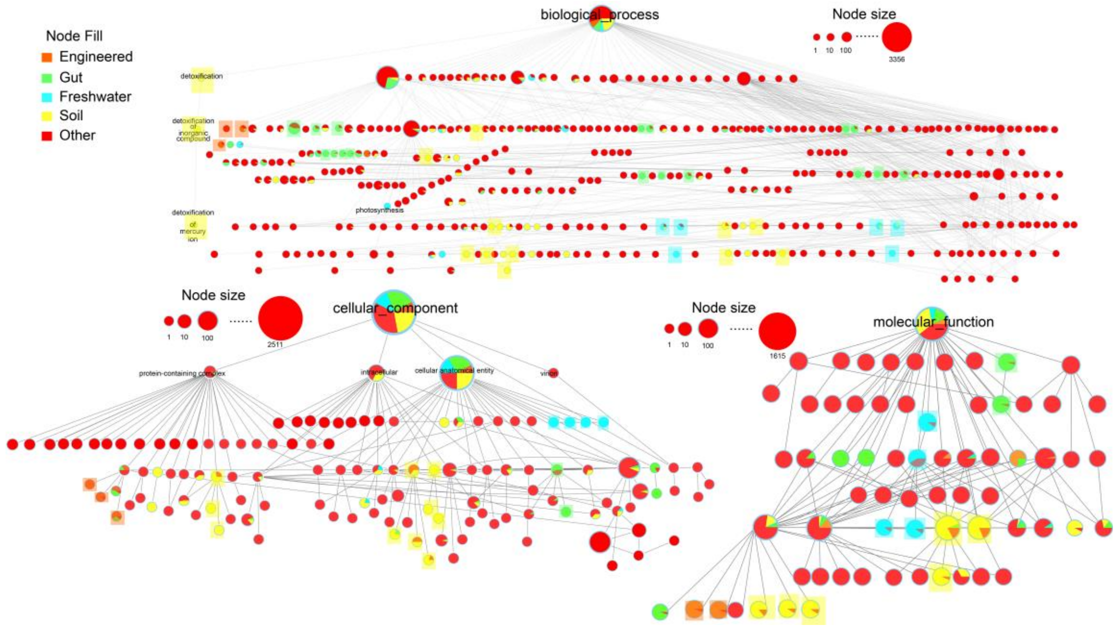

Figure S7. The function enrichment in four biomes based on the GO annotation. The GO annotation enriched in the specific biome (enrichment analysis, P-value<0.01) was labeled with the corresponding background color. The proportion of four biomes in a GO annotation was labeled on the corresponding Go annotation in pie chart form.

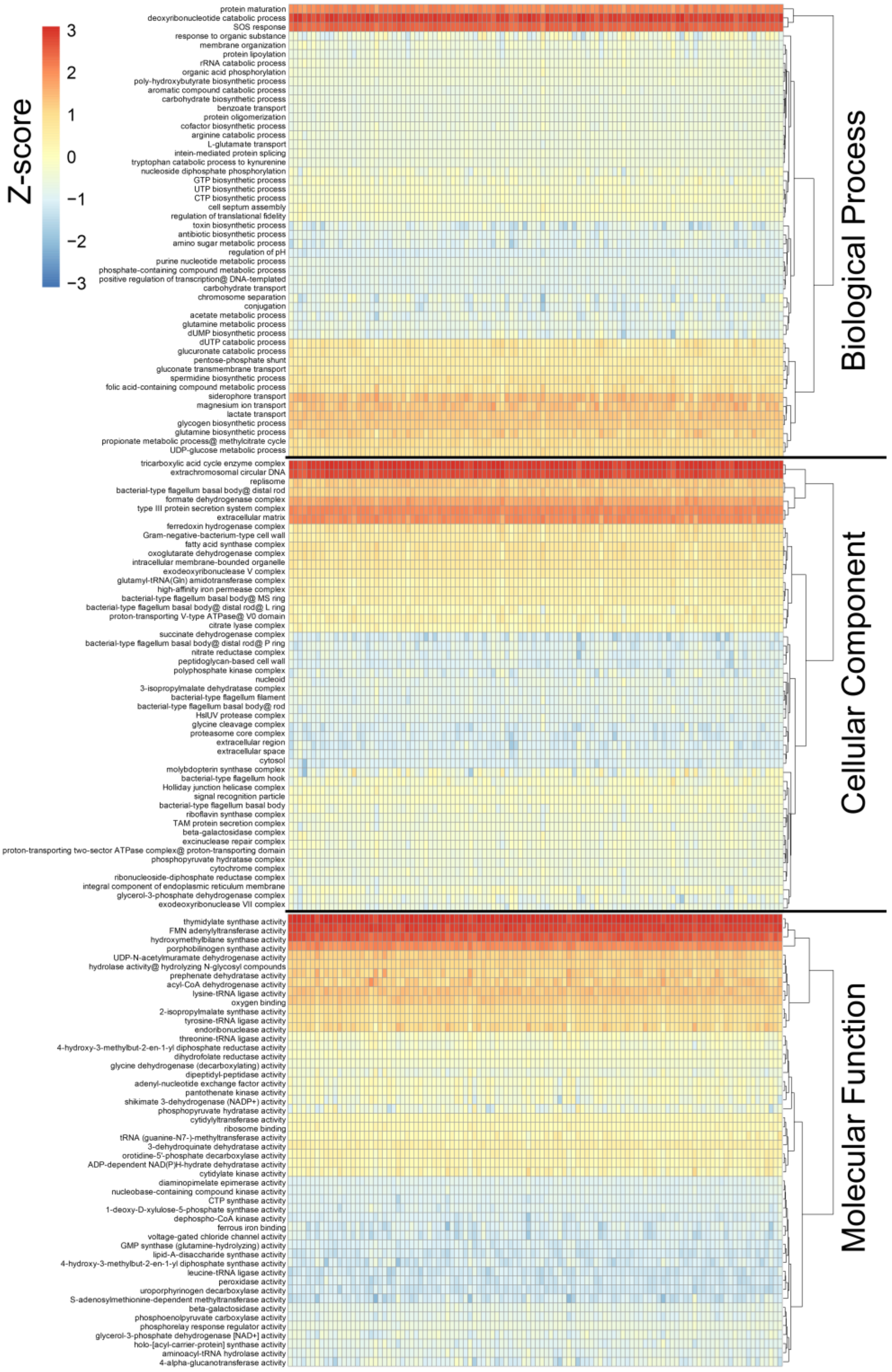

Figure S8. GO distributions for the Soil biome. The GO annotation was divided into three roots GO annotation (Biological Process, Cellular Component, and Molecular Function). Each row represents a GO annotation and each column represents a sample. For each root annotation, the top 50 GO annotation was shown, ranked by the number of counts. To compare among different samples, the number of GO annotations was normalized by samples using Z-score.

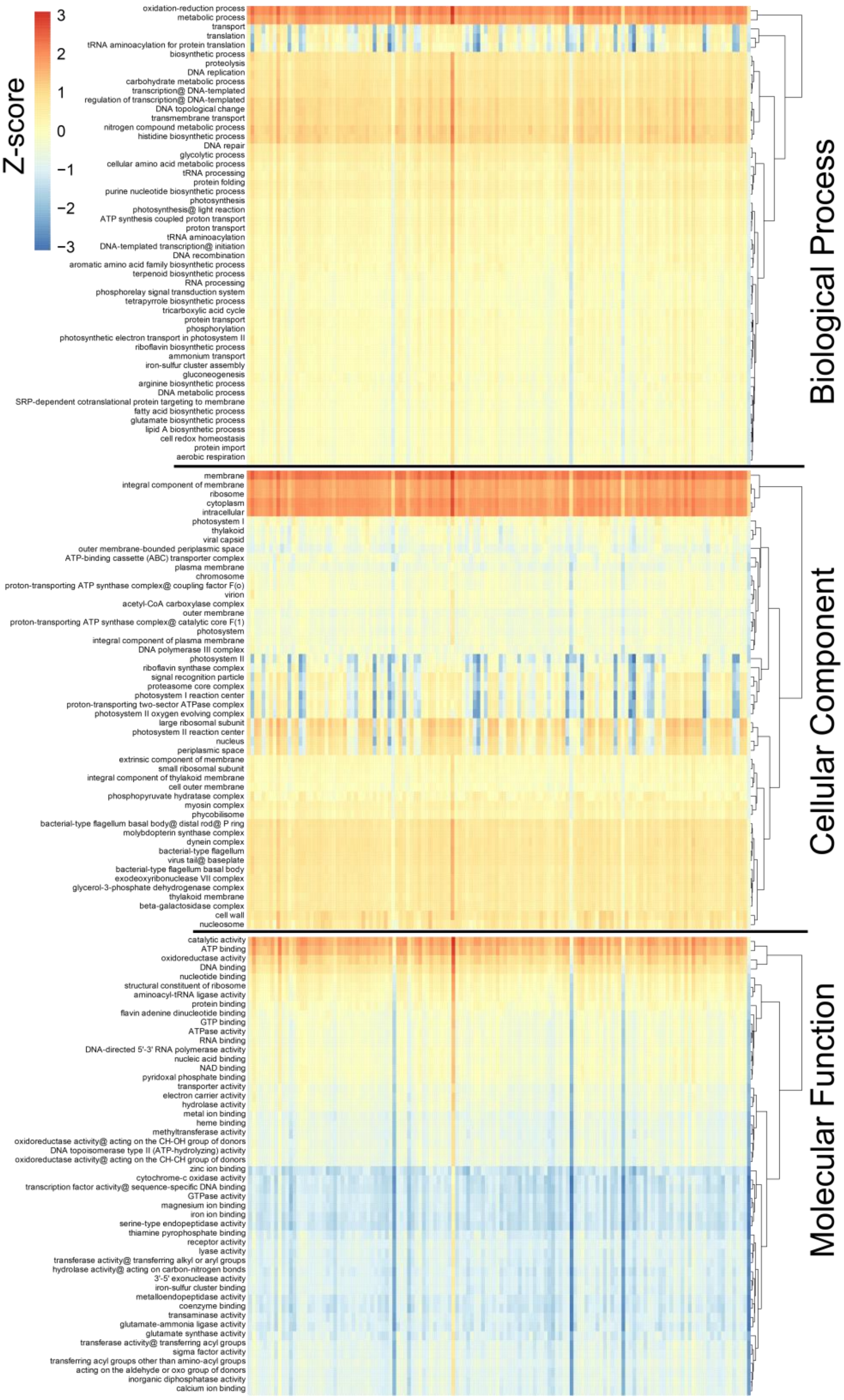

Figure S9. GO distributions for the Freshwater biome. The GO annotation was divided into three roots GO annotation (Biological Process, Cellular Component, and Molecular Function). Each row represents a GO annotation and each column represents a sample. For each root annotation, the top 50 GO annotation was shown, ranked by the number of counts. To compare among different samples, the number of GO annotations was normalized by samples using Z-score.

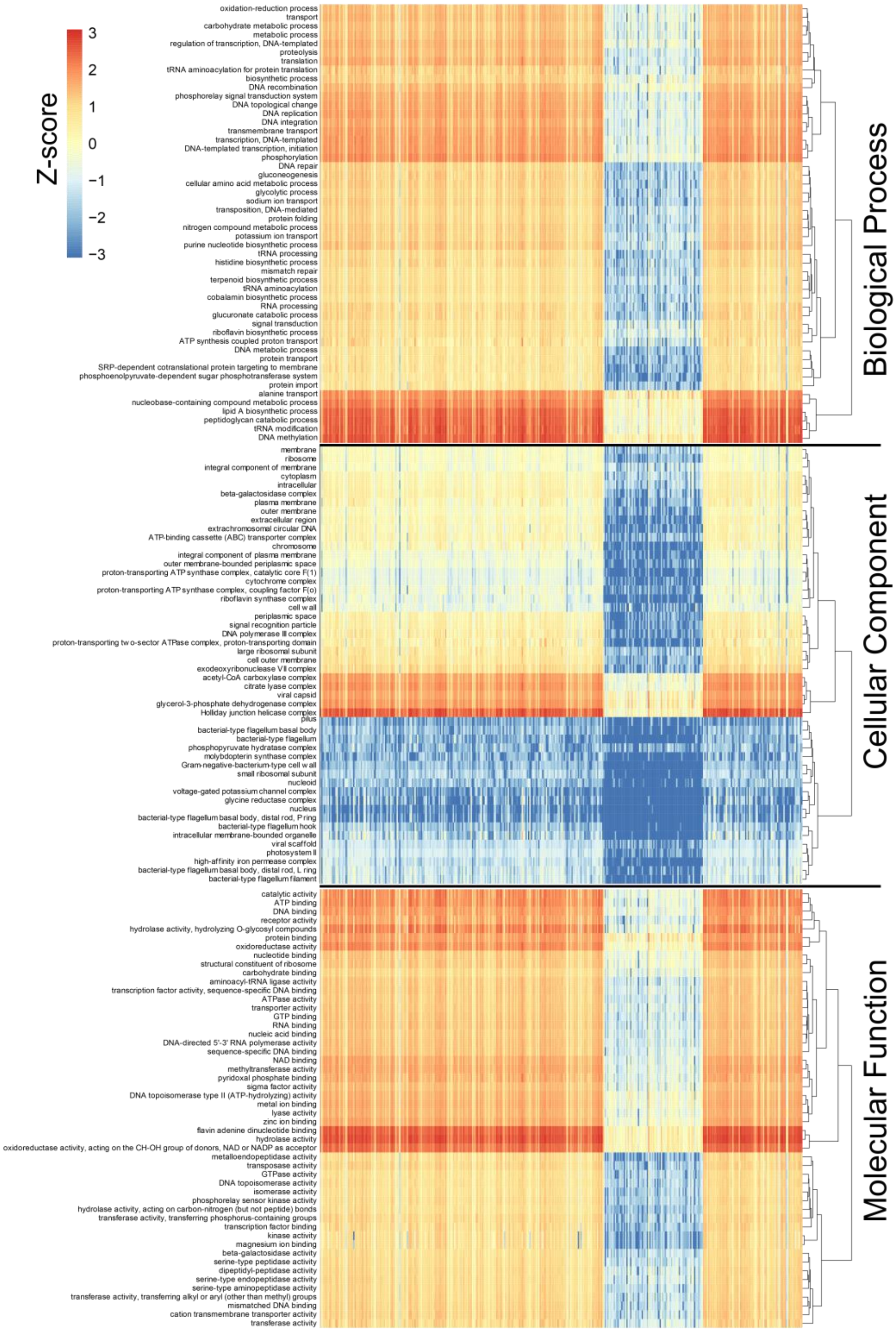

Figure S10. GO distributions for the Gut biome. The GO annotation was divided into three roots GO annotation (Biological Process, Cellular Component, and Molecular Function). Each row represents a GO annotation and each column represents a sample. For each root annotation, the top 50 GO annotation was shown, ranked by the number of counts. To compare among different samples, the number of GO annotations was normalized by samples using Z-score.

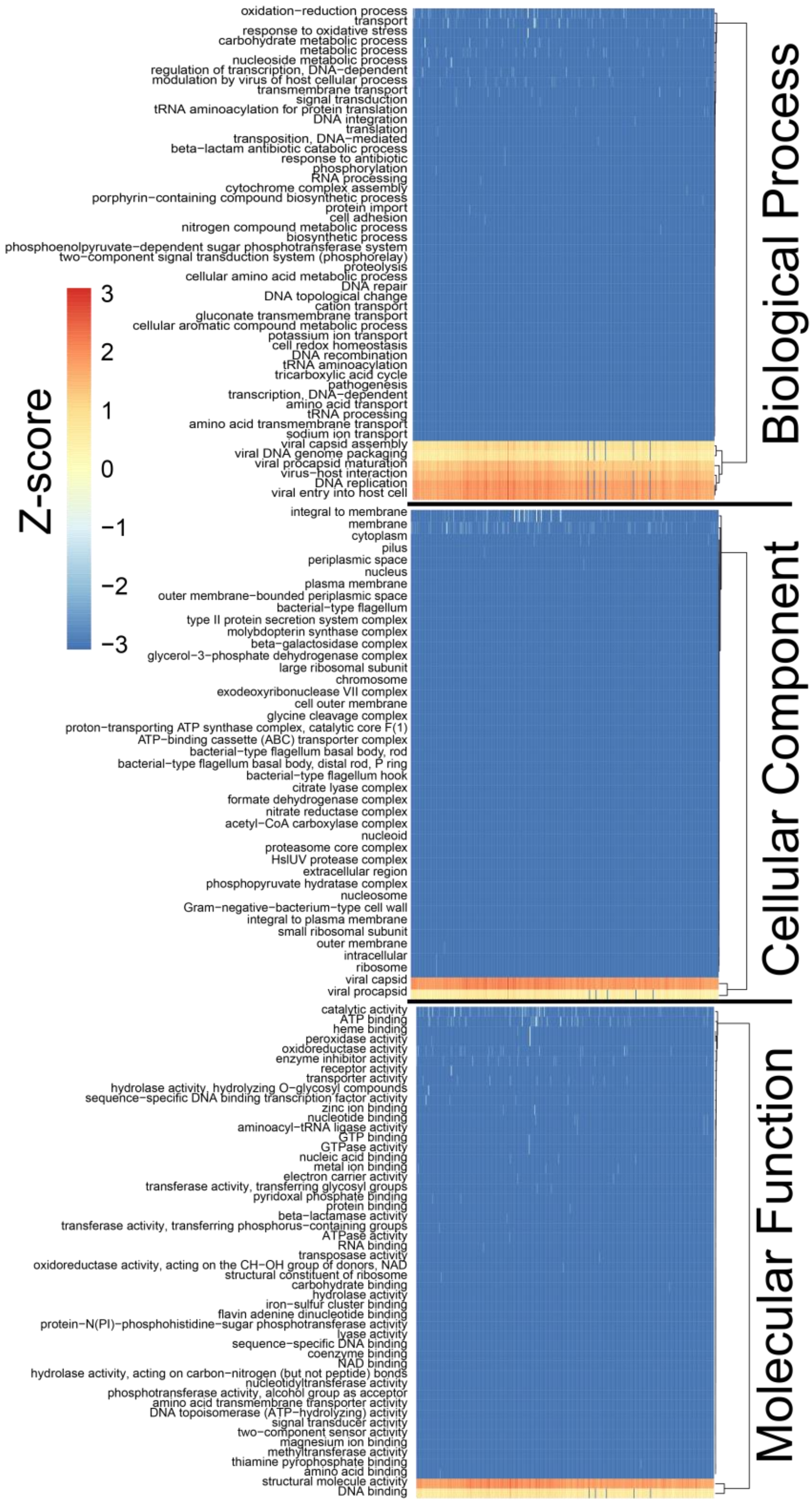

Figure S11. GO distributions for the Engineered biome. The GO annotation was divided into three roots GO annotation (Biological Process, Cellular Component, and Molecular Function). Each row represents a GO annotation and each column represents a sample. For each root annotation, the top 50 GO annotation was shown, ranked by the number of counts. To compare among different samples, the number of GO annotations was normalized by samples using Z-score.

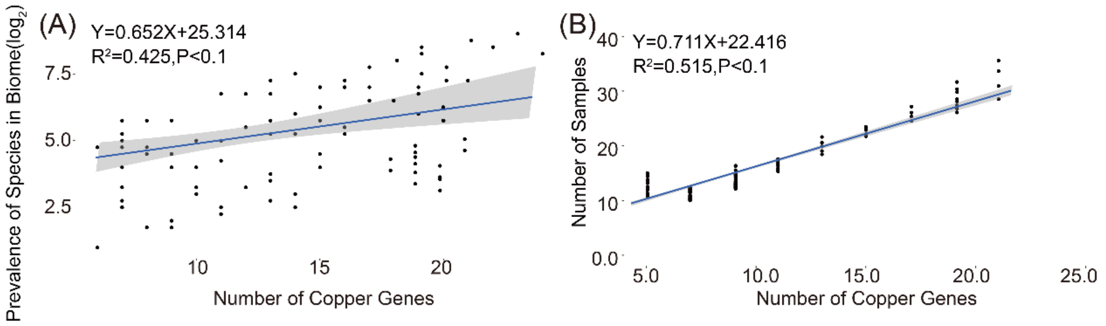

Figure S12. Correlation between the number of Copper genes and the prevalence of the host species in Soil biome. (A) Each node represents a species, and the X-axis represents the number of copper genes in that species. The Y-axis means the counts of species in the Soil biome. (B) The correlation between the proportion of samples containing a certain species (Y-axis) and the number of flagellum-related genes found in the Soil biome.

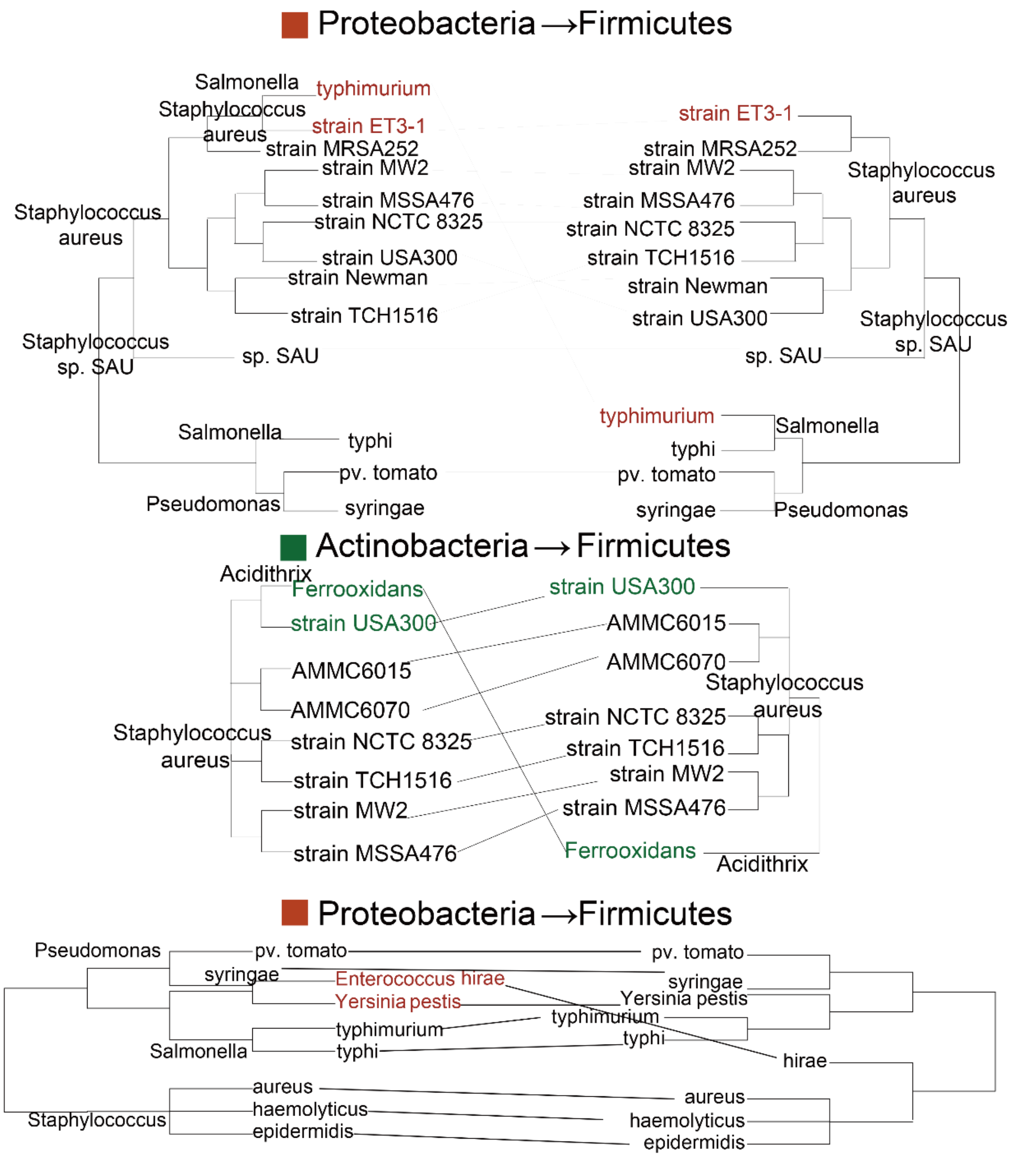

Figure S13. Phylogenetic tree constructed by copper gene family(left) and whole genome (right). This figure is supplementary to **Figure 3C**. To construct the phylogenetic tree based on HGT genes, all the sequences were aligned using Muscle (version 3.8.14), and the output format of multiple sequence alignment was set as Clustal. Then, the Clustal omega (version 1.2.4) was applied to construct a phylogenetic tree based on the multiple alignments. To compare the phylogenetic tree constructed by species and the HGT genes, R package ape (version 5.4) was applied, by calculating the number of stochastic dendrograms with equivalent or better Robinson–Foulds or matching cluster scores.

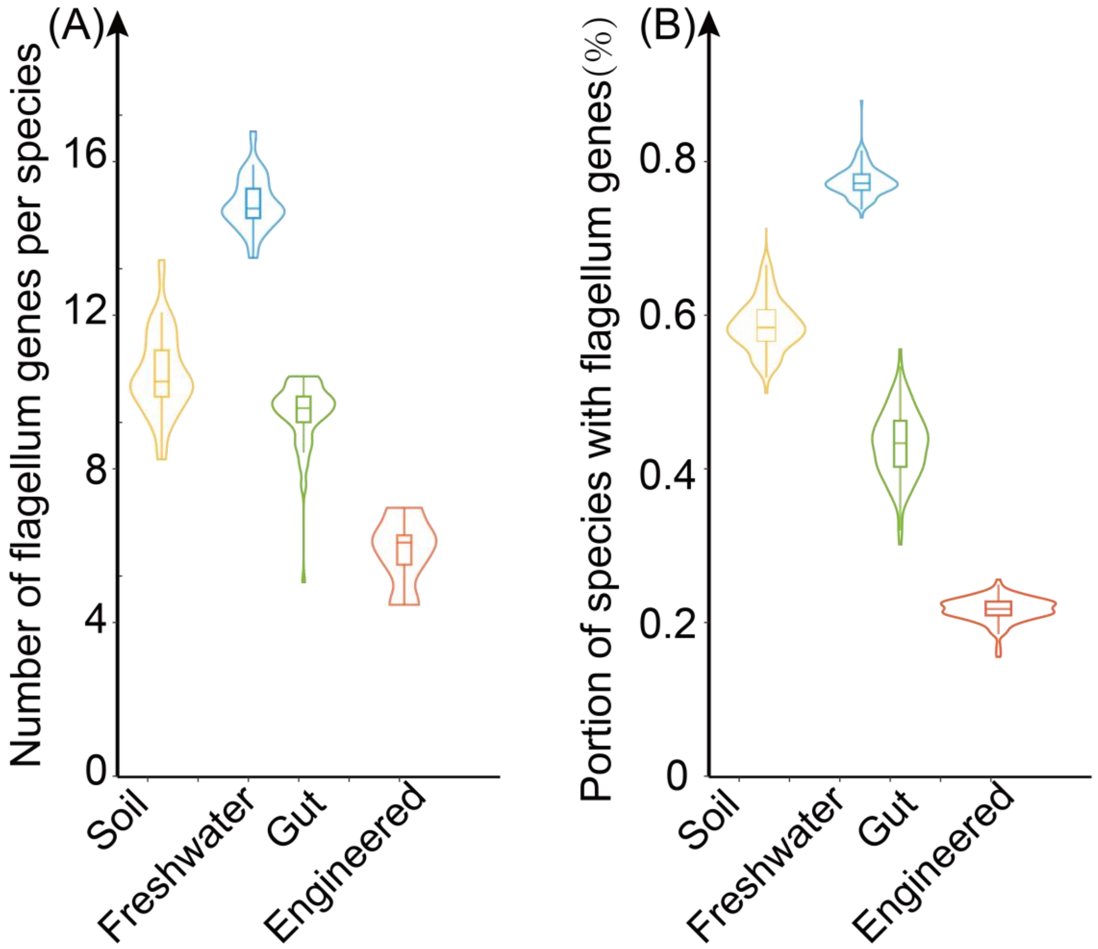

Figure S14. Statistic result of flagellum genes between four biomes. (A) the number of flagellum genes per species. For four biomes, the number of flagellum genes per species was 9.5 (Soil), 14.7 (Freshwater), 9.2 (Gut) and 7.3 (Engineered), respectively. (B) the portion of species with flagellum genes. For four biomes, the portion of species with flagellum genes was 0.57 (Soil), 0.78 (Freshwater), 0.48 (Gut) and 0.28 (Engineered), respectively.

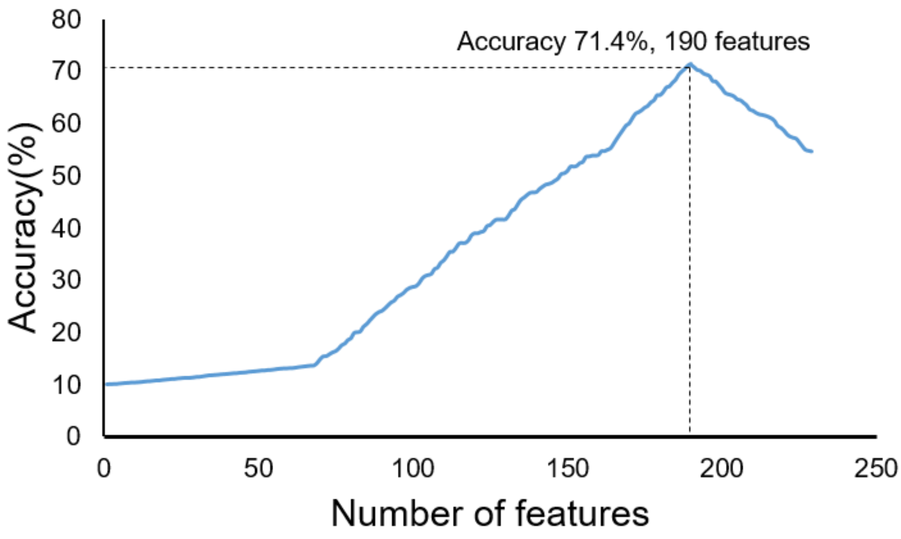

Figure S15. Feature selection process for random forest model. The X-axis represent the number of features in random forest model and the Y-axis means the accuracy of random forest model.

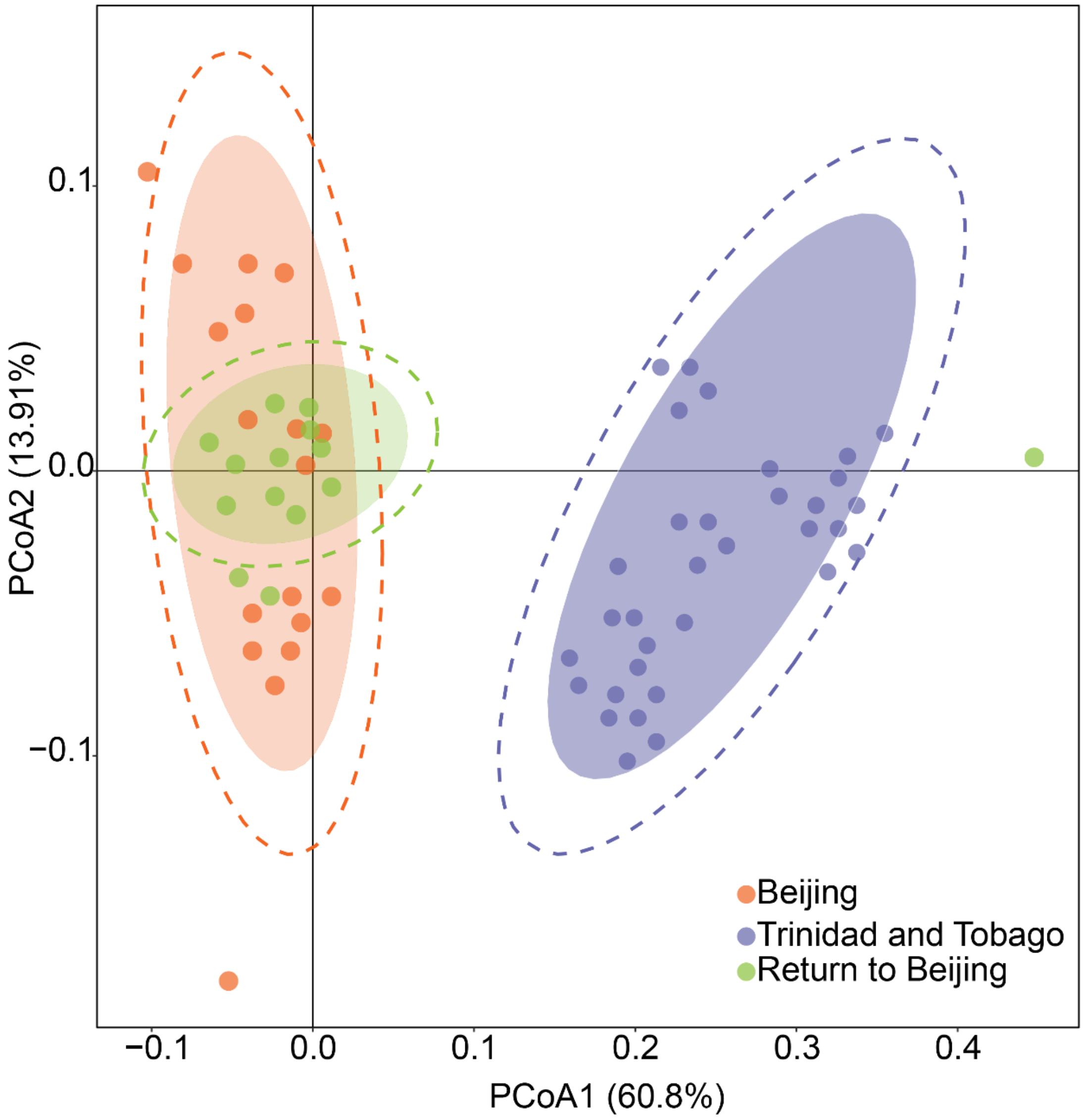

Figure S16. PCoA results based on the functional annotations from previous work (Liu H, *et.al.* Gut. 2019). This work recruited a Chinese volunteer team composed of 10 people who departed from Beijing, conducted a long stay of 6 months in Trinidad and Tobago and returned to Beijing. The PCoA result indicates that gut microbial communities are bidirectionally plastic and resilient across the long stay with multiple dietary shifts.

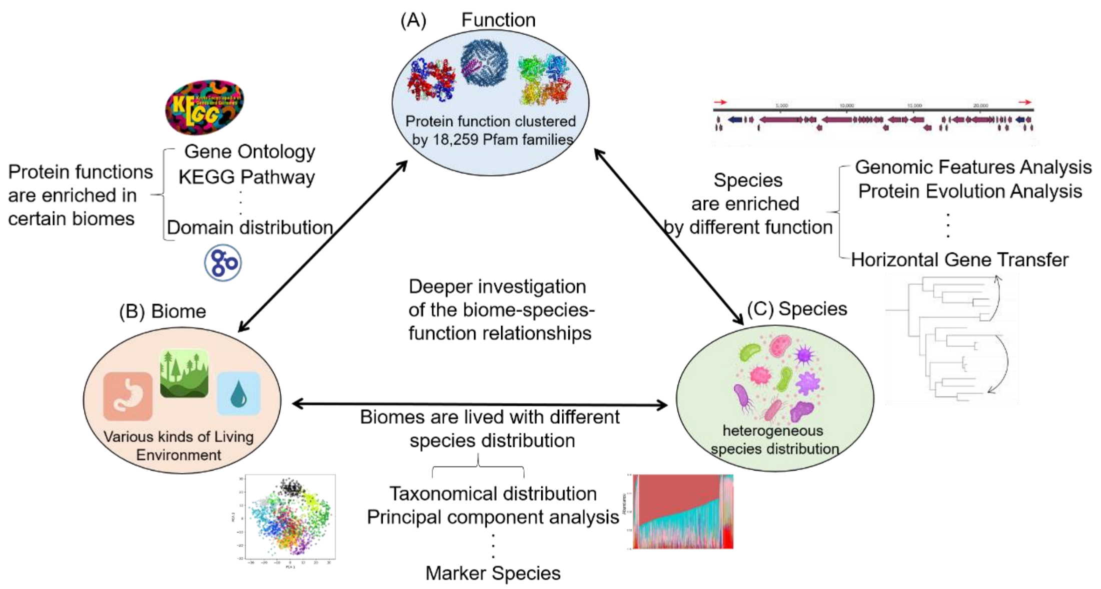

Figure S17. Workflow for the examination of the "biome-species-function" relationship. To explore the association between function(A) and biome(B), the gene annotation for functional genes in various biomes will be annotated by aligning to different functional annotation databases, such as the GO database, KEGG pathway. For the research of the association between biome(B) and species(C), a comprehensive set of comparative taxonomical analyses should be undertaken to determine the divergent species distribution in various biomes. For exploring the link between function(A) and species(C), the genome analysis is done to explore the function distribution for a genome.
