# Supplementary material for: Microbiome-based enrichment pattern mining has enabled a deeper understanding of the biome–species–function relationship
Source: Commun Biol. 2023 Apr 10;6:391. doi: 10.1038/s42003-023-04753-x (PMC10085995; doi:10.1038/s42003-023-04753-x)
Supplement: Supplementary file 2 — Description of Additional Supplementary Files [file 42003_2023_4753_MOESM2_ESM.pdf]

Description of Additional Supplementary Files

File name: Supplementary Data 1

Description: The list of GO annotation clusters and their host species for the "enrichment sphere" model.

File name: Supplementary Data 2

Description: The enriched GO annotations and their hosts selected for constructing the classification model.

File name: Supplementary Data 3

Description: The biomarker for four biomes based on the taxonomical compositions and functional profiles.

File name: Supplementary Data 4

Description: List of the project for four biomes (Soil, Freshwater, Gut, and Engineered) and related information.
