# Supplementary material for: Microbiome-based enrichment pattern mining has enabled a deeper understanding of the biome–species–function relationship
Source: Commun Biol. 2023 Apr 10;6:391. doi: 10.1038/s42003-023-04753-x (PMC10085995; doi:10.1038/s42003-023-04753-x)
Supplement: Supplementary file 7 — Reporting Summary [file 42003_2023_4753_MOESM7_ESM.pdf]

nature portfolio

Corresponding author(s): Kang Ning

Last updated by author(s): Mar 22, 2023

Reporting Summary

Nature Portfolio wishes to improve the reproducibility of the work that we publish. This form provides structure for consistency and transparency in reporting. For further information on Nature Portfolio policies, see our Editorial Policies and the Editorial Policy Checklist.

Statistics

For all statistical analyses, confirm that the following items are present in the figure legend, table legend, main text, or Methods section.

n/a	Confirmed	
☐	☒	The exact sample size (n) for each experimental group/condition, given as a discrete number and unit of measurement
☐	☒	A statement on whether measurements were taken from distinct samples or whether the same sample was measured repeatedly
☐	☒	The statistical test(s) used AND whether they are one- or two-sided<br>*Only common tests should be described solely by name; describe more complex techniques in the Methods section.*
☐	☒	A description of all covariates tested
☐	☒	A description of any assumptions or corrections, such as tests of normality and adjustment for multiple comparisons
☐	☒	A full description of the statistical parameters including central tendency (e.g. means) or other basic estimates (e.g. regression coefficient) AND variation (e.g. standard deviation) or associated estimates of uncertainty (e.g. confidence intervals)
☐	☒	For null hypothesis testing, the test statistic (e.g. F, t, r) with confidence intervals, effect sizes, degrees of freedom and P value noted<br>*Give P values as exact values whenever suitable.*
☐	☒	For Bayesian analysis, information on the choice of priors and Markov chain Monte Carlo settings
☐	☒	For hierarchical and complex designs, identification of the appropriate level for tests and full reporting of outcomes
☐	☒	Estimates of effect sizes (e.g. Cohen's d, Pearson's r), indicating how they were calculated

Our web collection on statistics for biologists contains articles on many of the points above.

Software and code

Policy information about availability of computer code

Data collection	No software was used in data collection. All data used was publicly available in an easy-to-download format.
Data analysis	All data was analyzed using the MGnify database. The data that support the findings of this study are all openly available. Supplementary Data 4 contains the accession numbers for all the metagenomes used. The codes used in our research were available at https://github.com/HUST-NingKang-Lab/biome-species-function-relationship.

For manuscripts utilizing custom algorithms or software that are central to the research but not yet described in published literature, software must be made available to editors and reviewers. We strongly encourage code deposition in a community repository (e.g. GitHub). See the Nature Portfolio guidelines for submitting code & software for further information.

Data

Policy information about availability of data

All manuscripts must include a data availability statement. This statement should provide the following information, where applicable:

- Accession codes, unique identifiers, or web links for publicly available datasets
- A description of any restrictions on data availability
- For clinical datasets or third party data, please ensure that the statement adheres to our policy

The data that support the findings of this study are all openly available. Supplementary Data 4 contains the accession numbers for all the metagenomes used. The codes used in our research were available at https://github.com/HUST-NingKang-Lab/biome-species-function-relationship.

Field-specific reporting

Please select the one below that is the best fit for your research. If you are not sure, read the appropriate sections before making your selection.

☐ Life sciences ☐ Behavioural & social sciences ☒ Ecological, evolutionary & environmental sciences

For a reference copy of the document with all sections, see nature.com/documents/nr-reporting-summary-flat.pdf

Ecological, evolutionary & environmental sciences study design

All studies must disclose on these points even when the disclosure is negative.

Study description	We collected metagenome data from the European Bioinformatics Institute (EBI) database, which is an organized database according to the habitat environments (biomes). The first layer of this database is divided into three biomes: "Engineered", "Environmental" and "Host-associated". To cover the representative biomes on Earth, samples from four representative biomes (Fermentor, Gut, Lake, and Soil) under these three biomes were downloaded
Research sample	The biome "Fermentor" was selected as a representative biome for the "Engineered" biome; the biomes "Soil" and "Lake" were selected as representative biomes for the "Environmental" biome; the "Gut" biome that includes human and animal (mice, pigs, cattle) intestines were selected for the "Host-associated" biome.
Sampling strategy	We collected metagenome data from the European Bioinformatics Institute (EBI) database, which is an organized database according to the habitat environments (biomes)
Data collection	We collected metagenome data from the European Bioinformatics Institute (EBI) database
Timing and spatial scale	The biome "Fermentor" was selected as a representative biome for the "Engineered" biome; the biomes "Soil" and "Lake" were selected as representative biomes for the "Environmental" biome; the "Gut" biome that includes human and animal (mice, pigs, cattle) intestines were selected for the "Host-associated" biome.
Data exclusions	No data was excluded
Reproducibility	all attempts to repeat the experiment were successful
Randomization	Study did not include any interventions and thus the randomization (as used in clinical trials or intervention studies) was not appropriate for this study. We do not anticipate any bias which might be caused by the potential lack of randomization.
Blinding	Study did not include any interventions and thus the conventional blinding (as used in clinical trials or intervention studies) was not appropriate for this study. We do anticipate any resulting bias which might be caused by the lack of blinding.

Did the study involve field work? ☐ Yes ☒ No

Reporting for specific materials, systems and methods

We require information from authors about some types of materials, experimental systems and methods used in many studies. Here, indicate whether each material, system or method listed is relevant to your study. If you are not sure if a list item applies to your research, read the appropriate section before selecting a response.

Materials & experimental systems

n/a	Involved in the study	
☒	☐	Antibodies
☒	☐	Eukaryotic cell lines
☒	☐	Palaeontology and archaeology
☒	☐	Animals and other organisms
☒	☐	Human research participants
☒	☐	Clinical data
☒	☐	Dual use research of concern

Methods

n/a	Involved in the study	
☒	☐	ChIP-seq
☒	☐	Flow cytometry
☒	☐	MRI-based neuroimaging
